# Productive use of energy of women-owned micro-, small-, and medium-sized enterprises: Insights from food and textile businesses in selected African countries

**DOI:** 10.1016/j.heliyon.2024.e32313

**Published:** 2024-06-01

**Authors:** Djalila Gad, Pierluigi Leone

**Affiliations:** Politecnico di Torino, Department of Energy (DENERG), Corso Duca degli Abruzzi 24, Turin, Italy

**Keywords:** Productive use of energy, Entrepreneurship, Gender, Renewable energy, Energy access, Africa

## Abstract

This paper presents a descriptive study focusing on the productive energy use of women-owned micro-, small-, and medium-sized enterprises that operate in Africa's food and textile sectors. Through a multidisciplinary approach, combining primary and secondary data collection methods, and integrating quantitative and qualitative tools, this study examines the relationship between the gender-based ownership structure of enterprises (i.e., sole female, female-female, and female-male) and energy consumption patterns, including demand levels, carrier use, access type (on-grid or off-grid), and expenditure. Despite limitations in scope and sample size, the findings shed light on gender-specific productive use practices.

Findings show that female-owned businesses primarily rely on single or dual energy carriers, contrasting with female-male enterprises, which typically employ two or more energy carriers. Fuel usage varies among ownership structures, with diesel, biomass, and liquified petroleum gas being notable choices. Increasing diversity in ownership correlates with heightened awareness of energy metrics and monthly demand for electric and mechanical power, with some of the latter correlation also observed for thermal energy. Moreover, as ownership diversity increases, energy expenditure per kilogramme of production output decreases. Some sole female-owned enterprises surpass 100 USD/kg/month, female-female partnerships may reach 100 USD/kg/month, whereas female-male co-owned enterprises remain below 10 USD/kg/month.

Beyond contributing to understanding gendered productive energy practices, this research also emphasises the importance of gender mainstreaming in productive use and energy access interventions. It highlights the need for renewable energy solutions, capacity-building programmes, and further research to address efficiency and accessibility challenges faced by women entrepreneurs.

## Highlights

• High research potential for gendered productive use in African food and textile sectors.
• The food sector is dominated by grid and fuel use, and the textile sector by grid use.
• Female-owned enterprises use 1–2 energy carriers, while female-male ones use 2-4.
• Distinct fuel use is observed across different gender-based ownership structures.
• Energy expenditure declines with rising gender diversity in enterprise ownership.

**Table:** ABBREVIATIONS

AFSIA	Africa Solar Industry Association
ADB	Asian Development Bank
CCF	Clean Cooking Fund
DEP	decentralised electrification project
EUC	electricity user cooperatives
ESMAP	Energy Sector Management Assistance Programme
FCV	fragile, conflict, and violence
IEA	International Energy Agency
IRENA	International Renewable Energy Agency
ISIC	International Standard Industrial Classification of All Economic Activities
kg	kilogramme
kWh	kilowatt-hour
kWheq	kilowatt-hour equivalent
LPG	liquified petroleum gas
LSEE	local sustainable energy enterprises
MECS	Modern Energy Cooking Services
MSME	micro-, small, and medium-sized enterprises
MTF	Multi-tier Framework
NGO	non-governmental organisation
OECD	Organisation for Economic Co-operation and Development
OGS	off-grid solar
RQ	research question
RNFE	rural non-farm enterprises
SAGER	Sex and Gender Equity in Research
SDG	Sustainable Development Goal
UNDP	United Nations Development Programme
UNEP	United Nations Environment Programme
UN Women	United Nations Entity for Gender Equality and the Empowerment of Women
USD	United States dollars
WE	Women's Economic Empowerment
WMSME	women-owned micro-, small-, and medium-sized enterprises

## 1 Introduction

In Africa's evolving economic landscape, women entrepreneurs are increasingly attracting attention in their role as owners of micro-, small-, and medium-sized enterprises (MSME) [1]. Nevertheless, these entrepreneurs confront distinct challenges compared to their male counterparts, mainly linked to limited access to resources and opportunities [2]. This is often explained by certain gender roles and norms associated with women [3] as well as their limited access to safe, affordable, and available mobility, and their disproportionate time allocation to household chores and care-taking activities which reduces their ability to undertake productive use activities or participate in education, training, and capacity-building programmes [4]. Literature also highlights that due to women's restricted access to financial instruments, land, and productive resources, they are more likely to engage in minor income-generating activities in the informal sector. Women mainly pursue activities related to cooking and sewing and are rarely able to participate in more technical sectors. Women's presence in low-income generating activities without access to or investment in education and training to improve their technical, business-related, leadership, and digital skills creates a vicious circle that degrades them to informal and unpaid work.

At the intersection of social science and energy research, there is a prevailing hypothesis that enhancing women's access to energy can yield numerous advantages [5], while its empirical foundation is still lacking. Although there exists a significant body of literature highlighting that gender plays a critical role in the productive use of energy,^1^ the lack of sex- and gender-disaggregated data on energy use patterns in the productive use sector limits our understanding of how women entrepreneurs consume electricity and fuels for their business activities, ultimately preventing the design of gender-sensitive energy policies and programmes that could lead to a sustainable and gender-just energy transition [[5], [6], [7], [8], [9]]. Collecting sex- and gender-disaggregated data on energy use can help to develop tailored energy solutions, potentially resulting in increased productivity and profitability of women-owned businesses, promoting gender equality, and contributing to economic growth and development.

The present study addresses these knowledge gaps by focusing on women-owned micro-, small-, and medium-sized enterprises (WMSME) in different African countries. The unique contribution lies in offering deeper insights into the productive energy use of WMSME and their role as end-users of energy products and services across different business sectors. This study is guided by two overarching research questions (RQ).

• RQ 1: Which African countries and industry sectors present viable cases for assessing the productive use of energy of WMSME?
• RQ 2: What are the productive energy use patterns among WMSME, encompassing energy carriers utilised, access type (on-grid^2^ or off-grid), monthly electric, mechanical, and thermal demand, and energy expenditure? Additionally, are there disparities in productive energy use across different countries, industry sectors, and gender-based ownership structures of WMSME?

A combination of secondary and primary data collection techniques is employed to comprehensively address these research questions, integrating qualitative and quantitative methods. The literature review synthesises insights from peer-reviewed sources from scientific journals and grey literature. For the first research question, secondary quantitative data collection relies on reputable databases. The second research question is addressed through primary quantitative data collection using a structured questionnaire distributed to participants based on pre-defined selection criteria. Data analysis involves descriptive techniques to elucidate patterns and disparities in the productive energy use among women-owned enterprises.

This paper is structured as follows: It commences by delineating the research design (section 2), which includes the rationale behind the research focus, the methodological approach adopted, and ethical considerations. Subsequently, section 3 provides foundational frameworks that underpin this study, and is followed by a state-of-art analysis. The methods employed in the literature review are outlined, and the findings are systematically presented to facilitate the synthesis of relevant content. In section 4, attention is directed towards the first research question, identifying African countries and industry sectors with the potential for high uptake of women entrepreneurs as energy end-users. The methods utilised are delineated first, followed by the presentation of results. Section 5 addresses the second research question, evaluating the productive use of energy in Egypt, Ghana, Kenya, Malawi, Nigeria, Tanzania, and Tunisia across women-owned food and textile businesses. The methods employed are outlined initially, followed by a discussion of limitations and the presentation of study results, including insights into business profiles, legal status, and energy use patterns. Moving forward, section 6 engages in a discussion on the question of how gender in enterprise ownership correlates with the productive use of energy. Finally, section 7 concludes the paper and provides recommendations.

## 2 Research design

This section introduces the research design for investigating the productive energy use of WMSME across different African countries. Beginning with a rationale for the research focus, the need to address the underrepresentation of WMSME as end-users of energy products and services in energy research is highlighted. Subsequently, the research questions are formulated, and the methodological approach to address them is presented encompassing both secondary and primary, as well as quantitative and qualitative data collection techniques. The section concludes with ethical considerations that guided the study.

### 2.1 Rationale for research focus

Identifying potential end-users of energy products and services, assessing their preferences, technology, and energy carrier^3^ use, potential benefits, and opportunities for large-scale adoption, is crucial for policymakers and other relevant stakeholders aiming to facilitate low-carbon transitions [10]. Therefore, user-focused research plays a vital role in informing policy decisions. Integrating social science into energy research is essential to comprehensively understand the societal factors influencing productive use patterns. The decision to concentrate on WMSME as end-users of energy products and services is rooted in the limited scholarly attention directed towards this demographic within the energy sector [11]. A thorough literature review revealed that while existing research predominantly targets households, farmers, communities, and women spearheading clean energy ventures, micro and small-scale entrepreneurs have often been marginalised. However, these enterprises hold a potential to benefit from mechanisation and a transition to renewable energies, thereby augmenting their revenue streams and alleviating labour-intensive tasks. As discourse surrounding energy access begins to pivot towards productive uses, there arises a need for research that specifically addresses the energy needs of WMSME.

Two overarching research questions (RQ) underpin this study.

• RQ 1: Which African countries and industry sectors present viable cases for assessing the productive use of energy of WMSME?
• RQ 2: What are the productive energy use patterns among WMSME, encompassing energy carriers utilised, access type (on-grid or off-grid), monthly electric, mechanical, and thermal demand, and energy expenditure? Additionally, are there disparities in productive energy use across different countries, industry sectors, and gender-based ownership structures of WMSME?

### 2.2 Methodological approach

The research approach was developed to be suitable, adaptable, and efficient in gathering evidence pertinent to the research questions while considering constraints like resources, time, and cost [12]. Drawing from existing literature it was evident that a typical methodology in this domain involves conducting a literature review and incorporating a quantitative questionnaire, qualitative (key informant) interviews, and focus group discussions [5,10,[13], [14], [15], [16], [17], [18], [19], [20]]. Such a mixed-methods approach, combining quantitative and qualitative data collection offers a comprehensive understanding of the complex interplay between productive energy use, electricity access, and gender dynamics [5]. It can result in providing more nuanced insights into the gendered aspects of energy use and allows for the amplification of marginalised voices often overlooked in solely quantitative studies. These considerations guided the selection of the research design, including methods, and techniques to ensure comprehensive data collection and analysis.

• The quasi-systematic literature review gathered qualitative and quantitative insights from both peer-reviewed sources from scientific journals and grey literature, similar to the approach adopted by Ref. [21]. Qualitative insights offered depth by exploring the “how” and “why” behind certain assessed phenomena, while quantitative analysis enhanced understanding through measurable data, collectively enriching the present research [12,21]. A systematic literature review was conducted utilising articles, book chapters, reviews, and conference papers sourced from Scopus, adhering to a rigorous and predefined protocol. This involved selecting and synthesising relevant studies about the research topic, following inclusion and exclusion criteria. Systematic data extraction and synthesis methods were applied, ensuring transparency and adherence to predetermined criteria at every stage of the review process. Additionally, the review was augmented by insights gleaned from grey literature sources, including reports, reviews, briefs, tip sheets, working papers, and guideline reports, following the same criteria for inclusion and exclusion. The identification of grey literature followed a non-systematic approach relying on the authors' familiarity with the research topic, consistent techniques were employed for inclusion criteria, data extraction, and synthesis methods. This ensured transparency and adherence to predetermined criteria throughout the review process.
• Secondary quantitative data collection was employed to address RQ 1 to enhance data reliability and replicability. Data was drawn from reputable databases accessed last in January 2024 (i.e., MSME Economic Indicators Database 2019, the World Bank's Enterprise Survey, and IEA's Gender and Energy Data Explorer) [[22], [23], [24]].
• Primary quantitative data collection for RQ 2 occurred between January 2023 to January 2024. Given the specific pre-determined enterprise selection criteria and the limited accessibility to participants, a deliberate and convenience sampling approach was employed [12]. Data collection was facilitated through the distribution of a structured questionnaire. This method mitigated interviewer bias and ensured respondents had adequate time to provide their answers. While this approach offered advantages such as widespread geographic coverage, it also presented challenges including inflexibility and the potential for ambiguous responses. The quantitative data were analysed using descriptive analysis which involved coding, editing, and tabulating the data, followed by drawing conclusions and interpreting the results to elucidate patterns and implications.

**Fig. 1:**
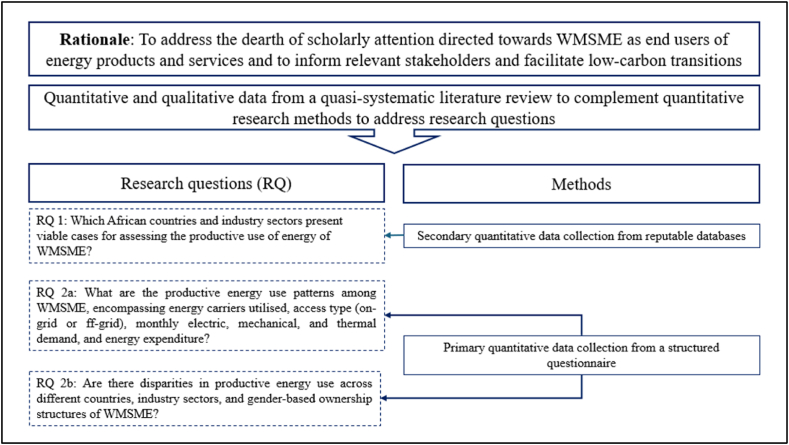
Adopted research design to address the underrepresentation of WMSME as end-users of energy products and services in energy research.

The research design is illustrated in Fig. 1.

### 2.3 Ethical approval

Data collection for RQ 2 involved a web-based questionnaire ensuring a comprehensive understanding of the participants' energy practices while upholding ethical standards and confidentiality. Verbal informed consent was obtained from all participants, reinforcing transparency and integrity. Approval from the ethical committee was not sought due to the minimal risk involved, the voluntary nature of participation, and stringent measures to anonymise data. Conducting research across diverse sectors and geographically dispersed regions made obtaining written consent impractical. Verbal consent, facilitated by trusted third parties such as governmental entities, intergovernmental organisations, universities, and industry associations, who connected us with the research participants, added an extra layer of privacy. Participants were assured of anonymity and willingly provided informed consent for publication, comprehending the objectives of the research study, and agreeing to publish their anonymised case details. Participants were informed about their rights and the handling of their data and were allowed to withdraw from the study at any time without consequences.

### 2.4 Ethical considerations

The web-based questionnaire, conducted via Google Docs, was designed in the form of a structured questionnaire, adhering to specific guidelines aimed at enhancing data collection [12,25,26]. The questionnaire was initiated with a concise introduction, inviting participants to fill out the questionnaire, and expressing gratitude for contributions. Questions were structured with answer choices and short-answer options for clarity and ease of understanding. Explanatory notes were thoughtfully incorporated where deemed necessary to enhance comprehension without introducing any form of bias. Technically, the questionnaire supported various platforms and browsers, saved progress made in the questionnaire, and collected both quantitative and qualitative responses. Upon completion, participants received a message thanking them for their involvement.

To safeguard privacy, options like “rather not say” were included for sensitive questions. Additionally, the invitation to the questionnaire was sent separately from the link to the questionnaire. The questionnaire was designed to prevent bias in question formulation, maintain consistent spelling, offer clear selection options, provide accurate instructions, define technical terms, allocate sufficient space for participants to answer open-ended questions, and utilise motivational techniques to encourage completion.

## 3 Understanding gendered energy dynamics: underlying theoretical frameworks and the current research status of productive energy use across the African continent

This section begins by delineating the foundational theoretical frameworks underpinning this research. It starts with examining the pivotal role renewable energies play in fostering sustainable development, alongside the concept of productive use of energy. Following this, a framework is outlined to elucidate the distinction between “sex” and “gender”. Subsequently, a quasi-structured literature review is presented to shed light on the prevailing research landscape surrounding productive energy use, paying particular attention to gendered perspectives within the context of productive female end-users across the African continent.

### 3.1 The role of renewable energies in fostering sustainable development

This research is guided by a theoretical framework that emphasises the endorsement of technological innovation, sustainable natural resource management, and economic development through the promotion of sustainable energies [27,28] in the focus countries under assessment. The policy recommendations and research directions outlined herein are deeply rooted in this overarching principle.

Acknowledging the intricate interplay among economic, social, environmental, and governance factors in directly and indirectly shaping sustainable development [[29], [30], [31], [32]], this study aligns with existing scientific research utilising the Environmental Kuznets Curve (EKC) hypothesis.^4^ Similar to Refs. [27,28,[30], [31], [32]], it underscores the significance of embracing eco-friendly technologies and cleaner energy sources, particularly in developing countries, to expedite their path toward sustainable development. However, it is important to note that while renewable energy consumption is often included as a variable in the EKC hypothesis, mixed results have been obtained, highlighting the nuanced relationship between renewable energy adoption and various environmental, social, and economic outcomes.

Nevertheless, the primary policy implication is to emphasise the substantial and accelerating impact of renewable energies on environmental sustainability [27,28], along with the potential positive socio-economic effects, particularly within the African entrepreneurial landscape. This paper contends that promoting renewable energy sources should involve fostering public-private partnerships and directing investments into renewable energy infrastructure [27,28,[30], [31], [32]]. A specific focus is placed on facilitating access to cleaner energy carriers for women-owned enterprises to enhance their productive activities. Providing substantial financial resources and establishing appropriate regulatory frameworks are imperative to support these partnerships and advance further research initiatives.

### 3.2 The concept of productive use of energy

Scholars and policymakers, along with various stakeholders, have employed the term “productive use of energy” in different contexts, and a consensus on its precise definition has not yet been found [10,33,34]. Existing definitions of productive energy use span across various dimensions. It can encompass different forms of energy carriers, including renewable, or sources defined as electric, and non-electric. Some definitions consider the end form in which energy is utilised, such as heat, mechanical power, or any other application. Productive use can also be defined by the activities undertaken post-energy supply, covering areas such as agricultural, non-farm, industrial, or commercial work. Many studies, especially in this research domain, primarily focus on the productive use of electricity and generally define it as the application of electrical energy services to activities aimed at income generation or productivity enhancement [33]. This concept is often articulated along three dimensions: improving the performance of existing income-generating activities, utilising electrical appliances powered by renewable energy sources (also in combination with off-grid energy supply), and fostering the creation of new enterprises and job opportunities post-electrification. While electricity-related factors are commonly recognised as pivotal drivers of productive energy use, it is often argued that they must be considered alongside other variables such as financial, technological, human capabilities, institutional frameworks, infrastructure, social dynamics, and demographic characteristics. Therefore, some researchers adopt a broader perspective, defining productive energy use as encompassing both electric and non-electric energy for activities aimed at enhancing income and welfare outcomes, promoting gender equality, improving health, and advancing education.

Similarly, in this context, productive use of energy is broadly defined as the utilisation of energy for direct income-generating activities, and for indirect benefits, like education, health, and improved gender equity outcomes [10]. In other words, the concept of productive use of energy generally adheres to Kapadia's definition, whereby the term refers to the application of energy, whether electrical or non-electrical in the forms of heat or mechanical energy, for tasks that improve income, overall well-being [35], and generate added value [36]. Embracing a broader, gender-sensitive view of productive use can facilitate the integration of gender considerations by various stakeholders involved in energy development, including public and private entities, civil society, researchers, funding institutions, and community members.

### 3.3 Distinction between “sex” and “gender”

To ensure precision and adherence to established academic guidelines, the Sex and Gender Equity in Research (SAGER) framework has been applied [37,38]. Within this framework, “sex” is defined as encompassing biological characteristics related to physical and physiological traits, including chromosomes, gene expression, hormone function, and reproductive/sexual anatomy, traditionally classified as female or male [37]. In contrast, the term “gender” extends beyond biological factors and binary classification (female/male), and encompasses socially constructed roles, behaviours, and identities that impact the allocation of power and resources within society. Within this research context, the use of “sex-disaggregated data” and “gender-disaggregated data” refers to information collected separately for both women and men, with the former adhering to binary categories (female/male) and the latter embracing a broader range of gender identities and expressions.

### 3.4 Literature review on gender-specific productive use of energy

This section provides an overview of the methods and techniques (3.4.1) employed in conducting a quasi-systematic literature review to gather insights for the research study. The approach involved integrating qualitative and quantitative data from both peer-reviewed sources from scientific journals and grey literature, ensuring a comprehensive understanding of the research topic. The presentation of findings (3.4.2) follows a structured approach, progressively funnelling from broader topics to a specific focus on women entrepreneurs.

#### 3.4.1 Materials and methods

The systematic literature review commenced with a search process utilising Scopus. Scopus was deemed more suitable than Web of Science as it aligns better with the research scope, covering modern materials significant to the relatively new research topic which dates back to the late 1970's [16]. While Web of Science offers a deeper historical search, Scopus provides a wide variety of publication fields and disciplines, including those in the humanities field, making it more comprehensive for the interdisciplinary research needed in this case. A predefined protocol was employed, whereby articles, book chapters, reviews, and conference papers were systematically sought, focusing on the intersection of productive energy use and gender. Boolean operators and wild card symbols were used to refine the search within relevant titles, abstracts, and keywords, and encompassed variations of “productive use” “energy” and “gender”. English language restriction was applied, resulting in 24 potential documents. The review adhered to stringent inclusion and exclusion criteria.

In addition to the systematic review, insights were gathered from a wide range of grey literature sources. Reports, reviews, briefs, tip sheets, working papers, and guideline reports from reputable international and regional institutions such as the International Renewable Energy Agency (IRENA), ENERGIA, various United Nations entities, RES4Africa Foundation, the World Bank Group, Asian Development Bank (ADB), Africa Solar Industry Association (AFSIA), 60 Decibels, Practical Action, Organisation for Economic Co-operation and Development (OECD), as well as European and African networks, foundations, and institutes. Employing similar inclusion criteria, the search encompassed documents linking productive energy use with gender, yielding 69 potential documents.

Systematic data extraction and synthesis methods were applied to both peer-reviewed sources from scientific journals and grey literature publications. Each document was manually reviewed and coded based on key attributes, including title, document type, author/publisher, publication year, identifier, geographical focus, methodology, key messages, and pertinent aspects of the research context. This systematic approach ensured comprehensive coverage and facilitated the synthesis of findings.

**Fig. 2:**
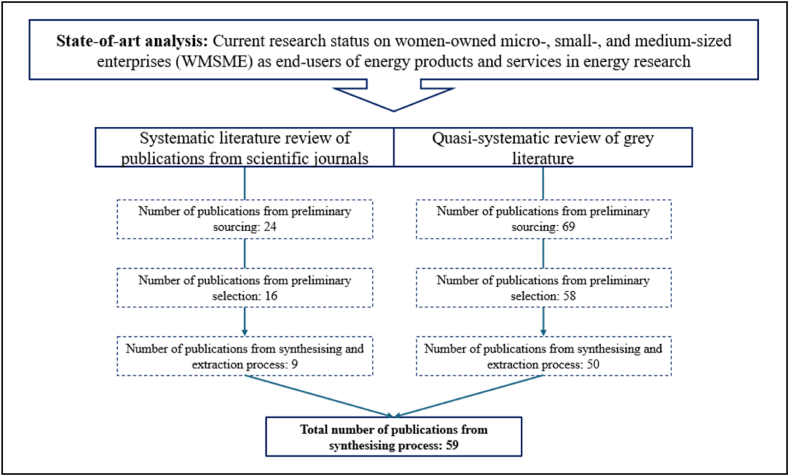
Number of publications from preliminary sourcing, and selection to synthesising and extraction process.

As demonstrated in Fig. 2, following an initial sourcing phase, the total number of documents decreased from 93 to 74, marking the preliminary selection phase. This phase comprised 16 peer-reviewed papers from scientific journals and 58 publications from grey literature sources, subjected to detailed assessment. Subsequently, 9 peer-reviewed papers from scientific journals and 50 grey literature publications were systematically synthesised, resulting in 59 documents. Their pertinent content was synthesised to align with the research rationale, and to address the research questions, laying the groundwork for the literature review and discussion in this research study.

The significant number of publications identified in grey literature indicates a notable interest in the discussion surrounding gender-specific productive energy use on the global stage. Moreover, there has been an observable upward trend in scholarly involvement with this topic in the past decade. A notable increase in scientific publications has been observed since the early 2000's, particularly after 2015, aligning with the agreement on the Sustainable Development Goals (SDGs). Notably, most peer-reviewed articles from scientific journals were published in 2020 and 2023. Within the grey literature, a similar pattern emerges, with a peak in publications in 2020. From 2012 onwards, there has been a consistent annual output ranging from 2 to 7 documents, with a notable increase observed from 2016 onwards. Geographically, the focus of publications aligns with regions where productive energy use is a prominent issue, namely in Africa, Asia, and Latin America. In the African context, the peer-reviewed literature from scientific journals predominantly addresses countries including Ethiopia, Ghana, Kenya, Nigeria, Rwanda, South Africa, Tanzania, and Uganda. Likewise, the geographic scope of grey literature publications across Africa encompasses Ghana, Kenya, Nigeria, Rwanda, South Africa, and Tanzania, with additional representation from Morocco and Senegal.

#### 3.4.2 Findings: assessing the intersection of productive energy use and gender, with a focus on women entrepreneurs as end-users

To systematically organise the literature review and facilitate the synthesis of pertinent content, thematic blocs were identified. Table 1 offers an overview of the subtopics utilised in the literature review process, and provides the respective number of documents addressing each topic.

**Table 1:** Thematic blocs for literature review and respective document counts. The sum of documents by thematic blocs will not equal the number of publications used for synthesis and extraction (which equals 59), as some documents cover more than one topic.

Subtopic for thematically structured literature review	Number of documents
Research context	5
Overlooked gender dimensions in energy research	5
Women in energy employment	13
Gender mainstreaming for enhanced energy access	16
Women in clean cooking	3
Impact of electricity on gender	3
Impact of electricity on women	6
Women promoting renewable energy access	11
Gendered productive use of energy	8
Women (entrepreneurs) as end-users of energy products and services	11

The literature review commences with an exploration of the research context and the often-overlooked gender dimensions in energy research. Subsequently, attention shifts to broader themes such as women in energy employment and gender mainstreaming for improved energy access, providing readers with an overarching understanding of the thematic landscape. While topics like women in clean cooking and the impact of electricity on gender are addressed by a few publications as per the adopted sourcing process, the role of women in promoting renewable energy access garners relatively widespread attention from the international community. Delving deeper, the gendered aspects of productive energy use are examined before concluding with an analysis of women entrepreneurs as end-users of energy products and services.

##### 3.4.2.1 Research context

While energy services for income generation may seem impartial to gender, the reality is starkly different [39]. A multidisciplinary approach challenges the prevailing view of electricity^5^ (or energy in general) as a gender-neutral force, highlighting its differential impact on men's and women's income generation potential [5]. Concepts like occupational segregation and social norms shed light on why women often show different energy use patterns as compared to their male counterparts [3,40]. Normative literature from donors and non-governmental organisations (NGOs) guides integrating gender perspectives into energy and entrepreneurship interventions. However, various challenges remain, and it is argued in the literature that female entrepreneurs encounter more obstacles in accessing energy carriers and services compared to their male counterparts [39]. Challenges include the informal nature of female enterprises and their limited access to complementary inputs, such as unequal access to financing for energy assets [3,5,40,41]. Societal norms often confine women's enterprises to home settings, potentially limiting their access to available and easily accessible energy carriers. Decision-making regarding energy assets, including appliance purchases like refrigerators and air conditioners, tends to be dominated by men, with many financial institutions still demanding male spouses to act as guarantors before extending financing to women. Demonstrating the efficiency gains resulting from women's productive use could offer an economic justification for integrating gender considerations into mainstream practices and could foster broader acceptance within the energy sector [5]. Further recommendations include recognising the varied roles of men and women in energy usage to increase targeted interventions, enhancing inter-agency coordination for more effective energy infrastructure planning, and developing specialised training programmes for women [3,40]. Additionally, there is a call for gender-sensitive financing policies and initiatives to offer women viable energy asset financing options. Providing women entrepreneurs with energy services tailored to their needs could serve as a foundation for operation, enhance sustainability, and offer them greater control over business operations [41].

##### 3.4.2.2 Overlooked gender dimensions in energy research

Recognising the women-energy nexus began in the late 1970's, focusing on challenges in gathering fuel wood for cooking [16]. Existing gender-energy research has primarily focused on women's challenges with accessing sustainable and reliable energy carriers and technologies for their reproductive roles within households [17]. Most research agrees that electrification boosts women's employment and shifts them away from agriculture, yet the quality of these new roles and their impact on earnings remains uncertain [17,42]. While some studies show electrification positively affects men's work hours, there is a lack of gender-specific insights into productive energy and fuel use at the enterprise level [16,17,19]. This omission disregards evidence of a gendered division of both labour and varying energy consumption patterns and fuel choices. Some studies suggest that women's reliance on biomass as a primary fuel source often results in their needs being sidelined as initiatives and programmes predominantly prioritise providing or enhancing electricity access and utilisation. Other research merely touches upon energy types used for cooking, heating, and cooling without delving deeper into the sector's energy requirements [41]. The lack of adequate, affordable, and reliable energy supply can impede women's ability to operate their enterprises profitably and safely.

##### 3.4.2.3 Women in energy employment

As indicated by IRENA, within the global renewable energy employment sector (public and private), women constitute 32 % of full-time employees, surpassing the oil and gas industry average of 22 % [3]. Women's employment is highest in the solar photovoltaic (PV) industry constituting 40 % of the full-time workforce [43], and is relatively low in the hydropower, and wind sectors where women represent 25 % and 21 % of the workforce, respectively [44,45]. Women's participation in technical, managerial, and senior management positions within the renewable energy sector remains lower than in administrative positions, attributed to various barriers like gender role perceptions, hiring practices, and lack of transparent policies [3,9,43,[45], [46], [47]]. Obstacles like the glass ceiling effect, cultural norms, limited mentorship opportunities, workplace harassment, and sexist language further hinder career advancement [3,43,[45], [46], [47], [48]]. Proposed solutions for public and private energy companies entail gender mainstreaming, continued gender-specific data collection and analysis, creating supportive networks and mentorship programmes, enhancing education access, and workplace policies, as well as setting gender targets [3,9,[43], [44], [45], [46], [47],[49], [50], [51], [52]].

##### 3.4.2.4 Gender mainstreaming for enhanced energy access

In both papers from scientific journals and grey literature publications, gender mainstreaming is increasingly recognised as a cross-cutting theme that plays a pivotal role in ensuring the long-term sustainability and socio-economic impacts of energy access programmes and projects [7,[53], [54], [55]]. This includes off-grid renewable energy mini-grids as well as utility-scale energy projects [7,[53], [54], [55]]. Moving beyond a technology-centric approach to integrate gender perspectives into various aspects throughout the life cycle of a project requires cross-sectoral planning strategies and sex-disaggregated data collection [3,[6], [7], [8],^53^,[56], [57], [58]]. Gender mainstreaming for enhanced energy access is advocated to be carried out during the planning phase of energy access initiatives, encompassing policy and programme design. It should incorporate gender-specific risk and mitigation strategies, along with capacity-building initiatives, and gender-sensitive financing models. Specific proposed actions include establishing dedicated funding channels or quotas for women-led households and enterprises, facilitating access to training and skills development programmes, and promoting women-led enterprises as role models to challenge gender stereotypes [6,8,50,[56], [57], [58], [59], [60], [61]]. A study conducted in Nepal and Sri Lanka concluded that an integrated gender approach specifically within the water-energy nexus can have positive gender equality and social inclusion outcomes, particularly when electricity access is provided at tier 3 or 4 of the World Bank's Global Tracking Framework [55]. However, despite progress, gender considerations are often addressed inconsistently in energy projects, hindering the systematic integration and comprehensive monitoring of results [39]. Similarly, a paper assessing companies that specialise in providing an array of solutions for household energy needs and powering tools for small-scale enterprises revealed that these companies lack familiarity with available gender mainstreaming resources and do not follow a structured gender inclusion strategy [15].

##### 3.4.2.5 Women in clean cooking

As indicated by the World Bank's latest estimates, approximately 2.4 billion people lack access to modern cooking services,^6^ with around 2.1 billion projected to remain in “cooking poverty” until 2030, compelled to rely on traditional fuels and technologies for cooking [62]. Sub-Saharan Africa stands out with the lowest rates of access to modern cooking services, highlighting significant regional disparities in energy access [63]. In its efforts to promote clean cooking initiatives, a World Bank study found that female primary cooks in households using biogas reported spending less time on cooking tasks and expressing greater satisfaction with available leisure time [62]. Underscoring the transformative potential of clean cooking technologies in alleviating burdens traditionally shouldered by women, the World Bank stresses the importance of recognising women as both beneficiaries and agents of change [64]. However, assessing the gender impact of clean cookstove adoption requires further development [62,64]. Gender considerations have often been overlooked in the design and evaluation of such initiatives. New instruments are required for measuring time-use agency, enhancing the feasibility and cost-effectiveness of monitoring co-benefits [62,64]. To accelerate the transition to clean cooking by 2030, the World Bank's Energy Sector Management Assistance Programme (ESMAP) launched the $500 million Clean Cooking Fund (CCF) in 2020 to increase commitments and investments in the clean cooking sector [64].

##### 3.4.2.6 Impact of electricity on gender

Given the multifaceted impacts of electrification initiatives, particularly decentralised electrification projects (DEPs), the literature suggests assessing their outcomes through various lenses [65]. While DEPs are often evaluated based on education and health outcomes, gender metrics, however, remain relatively underexplored. Insights from statistical analyses suggest that improvements in energy access through DEPs can have both positive and negative effects on gender dynamics, whereby the extent and direction of these effects may vary depending on the specific context and factors involved. Moreover, electrifying income-generating activities holds the potential to boost overall incomes, yet evidence suggests that women, who often work in less energy-intensive sectors, may not reap equal benefits compared to men [21]. This gendered impact is further exemplified by community-based micro-hydropower projects in Ethiopia [18]. While there were positive socio-economic impacts, women's specific energy needs and challenges were inadequately addressed. Despite appreciating the flexibility in time provided by electricity access, women experienced limited benefits from new income opportunities or leisure time, indicating a restricted recognition of their productive use activities.

##### 3.4.2.7 Impact of electricity on women

Grey literature cautiously suggests that access to time-saving electrically powered appliances would enable women to pursue work outside the home, reduce agricultural labour, and boost their participation in formal employment [[21], [66]]. When women's income-generating activities are electrified, the resultant productivity improvements can have broader impacts on women's lives. Some studies show that providing access to electricity for irrigation and processing equipment such as passive solar PV pumping and irrigation systems, increases the economic independence of women [[21], [67]]. Similarly, the absence of reliable and affordable electricity, particularly in fragile, conflict, and violence (FCV) settings disproportionately impacts women's entrepreneurial endeavours and their capacity to earn income [68]. They frequently engage in informal occupations like tailoring, cooking, and hairstyling, where electricity plays a vital role. These findings align with other research leveraging extensive data from electrified and non-electrified settings [66]. Although involving women in the provision of electricity and motive energy can enhance their well-being, the evidence supporting this notion is still limited [[21], [66]]. In that regard, a recent scholarly study, conducted in Nepal, indicated that women expressed optimism about business prospects post-electrification, but actual electricity usage for productive activities remained low [13]. Female employment saw no rise, and the role of electricity in business operations remained minimal. Similarly, another study investigated the nuanced impact of electrification on women in rural China, revealing that while rural electrification enhanced the perceptions of their social status, it did not lead to increased non-agricultural waged labour for women [69].

##### 3.4.2.8 Women promoting renewable energy access

The narrative around incorporating a gender perspective into energy access recently started to emphasise the crucial role of women in off-grid renewable energy implementation [43,46,70,[21], [71]]. Hereby, the increasing involvement of women in selling energy services, maintaining systems, and securing financing are highlighted [46,[21], [67],^70^,^71^]. Numerous examples from programmes involving cookstoves, hydro, and solar PV, highlight the essential role of women in delivering energy access solutions. Women, leveraging their networks and community ties, play a vital role in reaching the last mile. For instance, Solar Sister has trained 1200 women entrepreneurs who market and sell off-grid solutions, benefiting over 200,000 people to date [72]. Similarly, the Women's Economic Empowerment (WE) initiative collaborates with 3730 women entrepreneurs to facilitate the delivery of energy services and promote productive uses of energy, benefiting over 1.7 million consumers [73]. Projects like EmPower: Women for Climate Resilient Societies, led by the 10.13039/100004420United Nations Environment Programme (UNEP) and the 10.13039/100004420United Nations Entity for Gender Equality and the Empowerment of Women (10.13039/100004420UN Women) in Bangladesh, Cambodia, and Vietnam, serve as examples of initiatives combining policy actions, pilot projects, and knowledge sharing to support women entrepreneurs in the renewable energy sector [67]. Likewise, the UN-led programme “Women's Entrepreneurship for Sustainable Energy” advocates for the establishment of local sustainable energy enterprises (LSEE), also known as decentralised energy service companies or cooperatives [74]. These LSEE entities operate with innovative business models that align energy service payments with the cash flows of consumers with low energy consumption and limited ability to pay.

Despite facing greater challenges compared to men, such as discriminatory norms and limited access to financing and resources, women-owned enterprises are growing at a faster pace globally and achieve greater satisfaction rates compared to their male counterparts [46,75]. To further support women entrepreneurs in advancing energy access, the literature suggests addressing barriers like gender role perceptions, challenges in gaining recognition, and unequal access to finance, assets, and other resources [[21], [29],^43^,^46^,^67^,^75^].

##### 3.4.2.9 Gendered productive use of energy

Recent scholarly efforts have aimed to study gendered productive use patterns and identify gender-related barriers [5]. It is recognised that existing literature on this topic has predominantly centred on gender at the household level and overlooked gender dynamics within enterprises [5]. Some crucial studies in this research field delve into how gender dynamics shape the productive use of energy and draw empirical insights from case studies in Tanzania, Ghana, and Myanmar [16,17,19,76]. Men are often found in sectors like agriculture and fishing, which require higher energy inputs, while women typically handle tasks related to food preparation [76]. This disparity is often attributed to sectoral segregation, driven by gender norms dictating “appropriate” income-generation activities for women, limited access to finance, education, and resources for women, as well as the challenge of balancing caregiving responsibilities with work obligations [19,76]. Even when women and men work in similar sectors, women tend to be engaged at the lower end of the production value chain with less mechanised ventures. For instance, in Tanzania, men primarily engage in selling crops and cultivating cash crops, whereas women focus on subsistence crop cultivation for household consumption. Similarly, in Myanmar, men occupy higher-value roles within the fishing industry, while women are relegated to lower-paid positions in fish processing. In terms of gender-differentiated energy carrier use, it has been observed that businesses owned by men tend to rely more heavily on electricity for activities such as welding, car repairing, and operating sawmills [6,16,17]. Enterprises owned by women on the other hand primarily use cooking fuels like charcoal, firewood, or gas for their operations [6,17,19,57,76]. Similarly, a study assessing nuanced gender differences in energy needs at rural non-farm enterprises (RNFE) in Ghana has shown that women primarily operate food vending businesses and rely on traditional solid fuels, while men dominate electrical, wood, and metal-based enterprises [16]. Another study suggests slight gender differences in energy choices, with men tending to diversify their energy carriers more than women [77]. Although gas availability varies by geography, a significant portion of female entrepreneurs in Ghana utilise it, whereas diesel usage is more common among men-owned enterprises [76]. The literature emphasises that the higher cost of gas and electricity could pose a barrier to their widespread adoption, particularly among informal businesses [77]. While affordability plays a crucial role, other structural factors such as the woman's education level and her decision-making authority regarding the procurement and utilisation of energy carriers also influence fuel selection. Furthermore, some papers underscore that women-owned businesses generally demonstrate lower electricity consumption, and reduced performance in profits compared to male-owned enterprises due to factors like unskilled manual labour, lack of registration, and the informal and unorganised nature of micro-, and small-sized enterprises in which women predominantly operate [19,57,76]. However, contrasting to these findings results from assessing the Ghanaian enterprise development programme suggest that electricity usage positively correlates with business profits for both genders, with women-owned enterprises showing higher profitability [17].

The literature generally argues that a lack of emphasis on clean cooking energy, coupled with a focus on electricity-related interventions, results in men being the primary beneficiaries of energy initiatives, leaving women at a disadvantage [6,76]. To address these disparities and support both men and women in maximising the benefits of productive use, several recommendations are proposed [57,76,77]. This includes improved access to finance and equipment tailored to the specific needs of male and female entrepreneurs. To promote fuel switching and enhance efficient resource management, it is recommended to provide energy management training and capacity-building [57,76,77]. It is further suggested to enhance the reliability and affordability of energy supply to minimise business disruptions while addressing other business constraints. It is emphasised that education and training can challenge traditional gender roles and promote gender equity in productive use. Finally, exposure to role models who have overcome traditional gender roles can inspire and encourage women to engage in male-dominated sectors.

##### 3.4.2.10 Women (entrepreneurs) as end-users of energy products and services

The narrative around women entrepreneurs as end-users of energy products and services is multifaceted, showcasing both opportunities and challenges in leveraging their productive use of energy [54]. Generally, the literature agrees that the adoption of end-use technologies across various sectors (such as agriculture, agri-food processing, dairy production, retail, and cooking) holds promise in enhancing the productivity of women-owned enterprises [54,71,78]. However, gender disparities persist in accessing productive assets, technology, and financing [54]. Gender plays a significant role in decision-making regarding appliance acquisition, often with women having less influence, even for products primarily used by them [46,78]. As men predominantly purchase and own appliances, data collection often reflects their perspectives [78]. Indeed, women make up only one-third of off-grid product end-users as per the database provided by Ref. [75]. Yet, grey literature highlights that women are significant consumers of off-grid technologies, particularly in households and businesses [46]. It is further argued that since women tend to be more sustainable consumers, preferring eco-labelled products and energy-efficient options, understanding how women benefit differently from off-grid energy technologies can uncover market insights and identify new end-user segments. The exploration of gendered consumer demand for productive use appliances reveals a nuanced landscape, characterised by both consistency and divergence [79]. In a survey assessing gendered consumer demand for productive-use appliances, participants were asked whether gender influenced end-use product preferences. The findings reveal that while the majority (64 %) indicated no inclination to adjust their rankings based on the end user's gender, a significant portion (36 %) expressed openness to considering gender as a factor influencing product purchase. Notable disparities emerge in the preferences for specific products. For instance, sewing machines and electric cookers rank high for women but considerably lower for men. Solar water pumps, favoured by men, contrast with the lower usage rates among women. Hand power tools, despite expectations, exhibit lower demand among women. These findings are complemented by insights from another survey on off-grid products, which reveals that women constitute 42 % of end-users of solar lanterns [75]. Moreover, in sectors like solar home systems and mini-grids, women's representation remains comparatively low (20–30 %), possibly influenced by decision-making dynamics in financing contracts.

Adopting a gender-lens approach involves investing in companies led by women and considering their needs when designing products [20,46,80]. For example, ensuring that the productive use of equipment provided to women is suitable for their physical requirements, such as height and strength, to enable comfortable usage [20,46,80]. Some open-access guidelines offer women practical guidance and resources on how to assess different renewable energy technologies, enabling them to make informed choices based on their specific needs and circumstances [4]. Increased capital flows to the renewable energy sector, competitive financial markets to drive innovation, and the provision of tailored financing options, especially in the off-grid solar (OGS) and clean cookstove sectors need to be further leveraged [[20], [21]]. Available examples of initiatives either supporting women as end-users or assessing the productive use of women-owned enterprises are exceedingly limited. One instance involved a project designed to empower women entrepreneurs by providing comprehensive support to overcome social and cultural barriers [60]. Spanning multiple districts and electricity user cooperatives (EUCs) in remote areas, the project equipped women entrepreneurs across diverse business activities with technical, managerial, and marketing skills, facilitated access to finance and markets and provided capacity-building for effective energy resource management. Another example emerged from an assessment of women micro-entrepreneurs, revealing their significant presence in rural or semi-urban areas and reliance on electricity-run and clean energy-powered technologies [11].

Findings from the literature review have underscored the pivotal role of tailored energy services in bolstering the growth and sustainability of women-owned enterprises [41]. These enterprises often adapt to energy-related constraints dictated by their location and individual circumstances, necessitating access to diverse energy carriers to support their multifaceted operations. Despite some limited examples, the overall research landscape lacks comprehensive initiatives and studies focused on the productive use of women entrepreneurs. Therefore, this paper is directed towards targeting the specific productive activities undertaken by women and assessing utilised energy carriers, access type (on-grid or off-grid), consumption levels, and expenditures. In doing so, this paper aligns with three Sustainable Development Goals (SDGs) – namely, energy access (SDG7), gender equality (SDG5), as well as economic growth, full and productive employment, and decent work for all (SDG8). Addressing these goals is considered crucial in the pursuit of achieving sustainable energy access for all.

## 4 Exploring opportunities: identifying African countries and industry sectors with potential high uptake of women entrepreneurs as energy end-users

The selection of African focus countries was guided by the objective of identifying geographic locations with the highest density of MSME per inhabitant, coupled with a significant presence of women-owned enterprises. This effort was based on the assumption of a potentially high adoption of productive energy use in those countries, or at the very least, to mirror nations with relatively significant needs for productive energy, particularly within women-owned enterprises. The industry sectors were chosen based on two primary criteria: Firstly, industries with a considerable representation of women-owned enterprises were selected to ensure the inclusion of relevant research participants and to gain valuable insights into the predominant energy use patterns among such businesses. Secondly, sectors with diverse energy service requirements encompassing thermal, electric, and mechanical demands were prioritised due to their potential for renewable energy adoption and associated business opportunities.

### 4.1 Materials and methods

The country selection was based on the MSME Economic Indicators Database 2019 which provides the most current available data on the number of formally registered MSME, national MSME definitions, employment data, and enterprise sizes across 199 economies worldwide [22]. As for the African continent, the following economies are, however, not listed and were not considered for this research: Central African Republic, Chad, Comoros, Democratic Republic of Congo, Republic of Congo, Djibouti, Equatorial Guinea, Eritrea, Guinea-Bissau, Mauritania, São Tomé and Principe, Seychelles, Sierra Leone, and Somalia. The MSME Economic Indicators Database 2019 represents secondary data gathered between July 2018 and December 2018 while the original data mainly stem from statistical institutes, ministries, international organisations, small business promotion agencies, and research institutions, which have been cross-checked for this research. The data are not always standardised across time and countries which may compromise data comparability and aggregation. All available African countries were ranked according to the highest number of MSME per 1000 people and the highest share of women-owned MSME. Sex- and gender-disaggregated data gaps were filled by using the World Bank database [81] and the Gender and Energy Data Explorer provided by the IEA [23]. However, different methodologies and definitions, as well as a variance in the year when the data were collected and published may compromise data comparability. The World Bank data indicates the percentage of firms with a woman among the principal owners for selected years [81], whereas the IEA provides insights into the gender (binary and nonbinary categories) of start-up founders considering the year of enterprise establishment [23].

To identify and select viable industries for this research a combination of findings from literature research, data obtained from the Enterprise Surveys published by the World Bank [24], and technical reports that underscored the feasibility of adopting renewable energy in specific sectors were employed [82,83]. The Enterprise Surveys conducted and published by the World Bank across all geographic regions assess business performance indicators of small, medium, and large companies through face-to-face interviews with firm managers and owners [24,84]. Depending on the selected focus country for this research, data was collected from between 523 and 3075 firms during the years 2013–2020. The surveys constitute a representative sample of enterprises in the non-agricultural formal private economy and include the manufacturing, services, transportation, and construction sectors whereby public utilities, government services, health care, and financial services sectors are excluded. To quantify the number of women-owned enterprises by sector, the indicators “[gend1] Percent of firms with female participation in ownership” and “[gend6] Percent of firms with majority female ownership” were taken into consideration. Although the year of data collection varies for each selected focus country, it can still be assumed that the indicators are comparable across the assessed countries, given that the data were published after 2006 which is the year since the World Bank followed the Enterprise Surveys Global Methodology.

4.2 Findings: identifying Egypt, Ghana, Kenya, Malawi, Nigeria, Tanzania, and Tunisia as focus countries and food and textile sectors as viable industry sectors.

The MSME Economic Indicators Database 2019 provides insights into the entrepreneurial activity of a country [22]. Out of all the assessed African countries, the following 10 countries ranked highest in terms of numbers of MSME per 1000 inhabitants, respectively (see Table 2): the Federal Republic of Nigeria (hereinafter called Nigeria), the Republic of Ghana (hereinafter called Ghana), the United Republic of Tanzania (hereinafter called Tanzania), the Tunisian Republic (hereinafter called Tunisia), the Republic of Malawi (hereinafter called Malawi), the Arab Republic of Egypt (hereinafter called Egypt), the Republic of Kenya (hereinafter called Kenya), the Republic of Senegal (hereinafter called Senegal), People's Democratic Republic of Algeria (hereinafter called Algeria), and the Kingdom of Morocco (hereinafter called Morocco).

**Table 2:** Country ranking in terms of the highest number of MSME per 1000 inhabitants.

Country	Year	Number of MSME				MSME/1000 inhabitants	Share of women-owned MSME
		micro	small	medium	total MSME		
Nigeria	2013	36,994,578	68,168	4,670	37,067,416	215.7	43.3 %
Ghana	2015	1,083,938	802,928	338,372	2,225,238	80.7^a^	31.6 %^b^
Tanzania	2012	3,074,736	88,150		3,162,886	64.4	53.9 %
Tunisia	2016	720,639	16,115	2485	739,239	64.1	49.5 %^b^
Malawi	2012	799,859^c^	167,872^c^	19,749^c^	987,480	61.3	46.0 %
Egypt	2017	3,431,649	421,386	8311	3,861,346	39.6	17.8 %^b^
Kenya	2016	1,438,109	110,938	11,480	1,560,527	31.4	31.4 %
Senegal	2016	400,540	4895	1,632	407,066	25.7	22.9 %^b^
Algeria	2017	1,035,891	21,202	3,196	1,060,289	25.7	15 %^b^
Morocco	2002	733,662	14,123	2,417	750,202	25.4	13.1 %^b^

a The primary source indicates that the number of MSME per 1000 people amounts to 19.7 instead of 80.7. However, the value is kept as per the MSME database.

b Since the MSME Economic Indicators Database 2019 does not provide data on the share of women-owned MSME for Algeria, Ghana, Egypt, Morocco, Senegal, and Tunisia, World Bank data were added indicating firms with female participation in ownership in these countries [81]. These data values represent the percentage of firms with a woman among the principal owners. Kindly note that the reference years of the World Bank data for Algeria (2007), Ghana (2013), Egypt (2016), Morocco (2007), Senegal (2014), and Tunisia (2013) do not match the years of the MSME Economic Indicators Database 2019 which may compromise data comparability.

c Values were taken from the primary source as indicated by the MSME Economic Indicators Database 2019.

In this research, the first seven top-ranking African countries were selected, excluding the three lowest-ranking countries, Senegal, Algeria, and Morocco, leaving Egypt, Ghana, Kenya, Malawi, Nigeria, Tanzania, and Tunisia as selected focus countries. Interestingly, the geographic focus on African countries, predominantly observed in both peer-reviewed publications from scientific journals and grey literature regarding gendered productive energy use, closely aligns with the ranking findings. However, it's noteworthy that Tunisia, Malawi, and Egypt, identified as research countries with a high research potential, are not specifically covered in the existing literature.

**Fig. 3:**
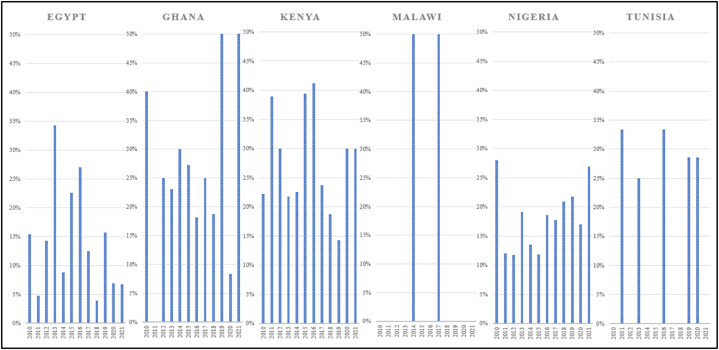
Gender-diverse enterprise founders (including women and other non-binary categories) across focus countries between 2010 and 2021.

All focus countries among the top 10 countries with the highest share of MSME per 1000 inhabitants, also rank highest in terms of share of women-owned enterprises, except for Egypt which ranks after Senegal [22]. To contextualise these figures, it is valuable to examine global and regional averages for a comparative analysis. Available data from the MSME Economic Indicators Database 2019 is too limited to provide global or regional averages of women-owned MSME, however, as per the latest data available from the World Bank, the global average of firms with female participation in ownership amounts to 33.3 %, the average for sub-Saharan Africa lies at 28.1 % while the average for Middle East and North Africa accounts for 19.0 % [81]. Additionally, the Gender and Energy Data Explorer from the Internal Energy Agency provides further insights [23]: between 2010 and 2021, the share of gender-diverse enterprise founders across the selected focus countries (except for Tanzania, since there are no data available) reaches a share of up to 50 %, ranging from less than 5%–34 % in Egypt, 0%–50 % in Ghana and Malawi, around 12%–28 % in Nigeria, 14%–41 % in Kenya, and 0%–33 % in Tunisia (see Fig. 3). This indicates that, except for Egypt, the selected focus countries consistently surpass either global averages or, at a minimum, regional averages.

The distinction between micro-, small-, and medium-sized enterprises differs across countries and is commonly indicated by the number of employees, assets, or turnover [22]. However, since most of the assessed countries only list data on the number of employees, this research refers to the size of MSME as per the respective country's distinction in terms of the number of employees, shown in Table 3.

**Table 3:** Country definition of MSME according to the number of employees.

Country	MSME definition (number of employees)			
	micro	small	medium	large
Egypt	1–4	5–49	50–99	>99
Ghana	1–9	10–30	31–100	>100
Kenya	1–9	10–49	50–99	≥100
Malawi	1–4	5–20	21–100	>100
Nigeria	1–9	10–49	50–199	≥200
Tanzania	1–4	5–49	50–99	>99
Tunisia	<6	6–49	50–199	>199

As per the MSME Economic Indicators Database 2019, and compared to small-, medium-, and large-sized enterprises, micro-sized enterprises have a prominent presence in all focus countries, accounting for 99.8 % in Nigeria, 97.5 % in Tunisia, 97.2 % in Tanzania, 92.2 % in Kenya, 88.9 % in Egypt, and 81 % in Malawi. In Ghana, the share of micro- and small-sized enterprises lies at 48.7 % and 36.1 %, respectively.

Literature highlights that women predominantly use energy for productive activities related to food processing, handicrafts, and weaving, which require energy to cover demands for lighting, cooking, and processing equipment [2,61]. The agri-food and textile industries are both sectors dominated by micro- and small-sized enterprises [4], which represent enterprise sizes that tend to show a higher share of women ownership compared to larger-sized firms [61]. The agri-food industry encompasses all activities related to the processing, preserving, preparing, packaging, and marketing of agricultural and food products [71], while the textile industry commonly includes the design, production, and distribution of yarn, cloth, and clothing products [82].

**Fig. 4:**
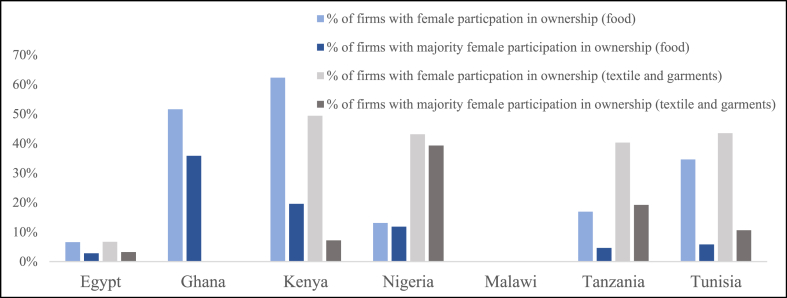
Share of firms with female participation in ownership across sectors and focus countries.

Quantitative data from the Enterprise Surveys published by the 10.13039/100004421World Bank supports the findings from the literature, indicating that the textile and food sectors across all assessed countries (where the data is available) show a considerable share of women-owned enterprises [24]. Fig. 4 shows the share of firms with (majority) female participation in ownership.

While data availability of most subsectors of the manufacturing and services sectors vary across the focus countries, the share of women-owned enterprises in the food sector and the textile and garments sector is available in most focus countries (except for Malawi) [24]. When compared to all subsectors, the food, and the textile and garments sectors consistently rank among the top three subsectors (alongside wholesale and retail) with the highest share of firms with female participation in ownership, except for Egypt. In Egypt, both, the food, and textile and garments sectors are positioned below other sub-sectors concerning female ownership participation following non-metallic mineral products, chemicals and chemical products, hospitality and tourism, petroleum products, plastics and rubber, and leather products. In Nigeria, the food sector follows several other sub-sectors (including garments, other services panel, retail, hotels and restaurants, manufacturing, and printing and publishing), but the garments sector takes the lead when it comes to female participation in ownership. It can also be noted that the share of firms with a majority of female participation in ownership ranks highest in the textile and garments industry in Nigeria (39.3 %), Tanzania (19.2 %), and Tunisia (10.6 %) while in Ghana the food industry ranks highest (35.8 %). In Egypt, the share of firms with a majority of female participation in ownership ranks second highest in the textile and garments sector (3.2 %) after non-metallic mineral products (8.3 %). In Kenya, the share of firms with a majority of female participation in ownership ranks second highest in the food subsector (19.6 %) after hospitality and tourism (21.5 %). Data on (majority) female participation in ownership is not available for Malawi.

In addition to the significant representation of women-owned enterprises in the food and textile sectors, it can be noted that there exists an opportunity for these MSME to embark on a transformative journey towards clean and sustainable energy alternatives [[71], [82]] which further solidifies the need for the present research. With relatively low-temperature heat demands ranging from 30 to 120 °C in the food sector, and from 30 to 180 °C in the textile sector, there exists a yet unfulfilled potential for MSME to make use of renewable energy sources like solar thermal [83], geothermal, biofuels, green hydrogen [85] or solar-driven heat pumps. Energy efficiency also plays a crucial role in the energy transition process of these industry sectors [82]. In addition, electricity requirements for productive uses in the food and textile sectors potentially entail the integration of renewable power-based options such as solar PV, or micro-hydro, among others.

## 5 A gender perspective: assessing productive use of energy in Egypt, Ghana, Kenya, Malawi, Nigeria, Tanzania, and Tunisia across women-owned food and textile businesses

This section presents a comprehensive examination of gender dynamics influencing the productive use of energy in food and textile industries across Egypt, Ghana, Kenya, Malawi, Nigeria, Tanzania, and Tunisia. Section 5.1 provides an overview of the materials and methods employed in the study. Following this, in section 5.2, the limitations of the study are discussed, providing transparency about the scope and constraints of the research. Building upon this foundation, section 5.3 delves into a descriptive analysis of energy use patterns among enterprises with female participation in ownership.

### 5.1 Materials and methods

To identify and engage women entrepreneurs for the present research, an extensive one-year-long outreach campaign was conducted from January 2023 to January 2024. This initiative aimed to connect with as many women entrepreneurs as possible within the designated focus countries. In each country, the outreach strategy involved contacting key entities responsible for collaborating with or having access to women entrepreneurs, potentially maintaining relevant databases. The primary entities involved in supporting women entrepreneurs included governmental and non-governmental organisations, industry associations, intergovernmental institutions, development organisations, and private companies operating in related sectors. Within the scope of the outreach campaign, it was communicated that enterprise selection adheres strictly to predetermined criteria and indicators established for the research design, similar to Ref. [16].

• The enterprise may be owned by a single individual but must qualify as an enterprise classified as micro-, small-, or medium-sized, with at least one female owner in the ownership structure.
• The selected energy form must be exclusively allocated for productive purposes, excluding community or household usage. The type and number of energy carriers were not a determining factor.
• The enterprise must operate within the food or textile sector as defined by the International Standard Industrial Classification of All Economic Activities (ISIC).

Indicators that aligned with the research objectives were translated into a quantitative survey [86], with each question designed to serve a specific purpose. This rationale encompassed identifying respondents and their respective roles within the enterprise, as well as capturing essential insights into the geographical and urban/rural distribution. Additionally, questions were developed to gather insights into ownership structures, enterprise sizes, legal statuses, business sectors, and specific activities. Moreover, the survey aimed to capture data on utilised energy carriers, energy access type (on-grid or off-grid), electric, mechanical, and thermal consumption levels, as well as energy expenditure regarding production output. Finally, the web-based questionnaire was categorised into two parts with 14 questions. On average, participants spent approximately 15 min completing the questionnaire. The first part captured enterprise-related information (i.e., location, gender of owner and co-owner (if any), size (number of employees), enterprise type (formal or informal sector), industry sector (food or textile), type of business activity, and annual production output), while the second part investigated energy-related information (i.e., energy carrier, average monthly electricity and fuel demand, and annual electricity and fuel costs). The questions were designed to be as simple as possible, and an explanatory note was included to assist participants in understanding and selecting their answer choices. An overview of the questions posed, type of answer choices, and notes (if applicable), as well as the rationale behind each question (which was not visible to the research participants), can be found in the Appendix, Table A1.

The web-based questionnaire, available in English and French, was created as a Google Doc form, and a link was shared with participants either via email or WhatsApp, depending on the communication method preferred by or available to the respective participant. Responses were collected for a whole year, from January 2023 to January 2024.

The questionnaire responses underwent a comprehensive evaluation process using Microsoft Excel software for data analysis, aligning with the methodology of a descriptive study. To ensure data accuracy and relevance, several steps were taken: each response was cross-checked against the predetermined criteria and indicators established for the research design (as outlined above). Specifically, only enterprises with at least one female owner and falling within the micro-, small-, or medium-size categories based on employee numbers were considered. Additionally, only businesses engaged in productive use activities in the food or textile sectors, as defined by ISIC, were included. Duplicated responses were systematically identified and eliminated.

The evaluation process encompassed the segregation of responses by country, and subsequent assessment to unveil productive use patterns categorised by industry sector and gender-based ownership structure. Businesses with female participation in ownership were classified into three categories: (i) enterprises owned solely by a female, (ii) enterprises characterised as a female-female partnership, and (iii) enterprises with co-owners who identified themselves as “female and male” or “other and male” or “female and not specified” (for simplicity, this third category is herein referred to as female-male only).

The process of estimating the average monthly electrical, mechanical, and thermal demands was grounded in a robust methodology that was developed to address the limited scope of the inquiry, which primarily aimed to identify the energy carriers necessary to meet productive needs. A comprehensive study was conducted on each enterprise individually, considering their business activity, production value chain, final production output, and energy carriers utilised. This led to the formulation of the following assumptions: It was presumed that electrical and mechanical demands would be met by a combination of grid electricity, solar PV,^7^ and diesel or petrol generators. In contrast, thermal demand was hypothesised to be satisfied by LPG, natural gas, kerosene, biomass, and energy carriers classified as 'other'. Following this analysis, all monthly consumed energy carriers originally provided in different energy units (i.e., kilowatt-hours, kilogrammes, and litres) were uniformly converted to kilowatt-hours equivalent (kWheq) per month [[87], [88], [89], [90], [91], [92]]. Similarly, all production outputs were converted to kilogrammes (kg) per month [[93], [94], [95], [96], [97]] while financial data, including energy expenditure, was converted to United States dollars (USD) using an average conversion factor spanning from January 1, 2022, to December 31, 2022 [[98], [99], [100], [101], [102], [103], [104]] (see Appendix B, Table B1 for applied conversion factors). These conversions facilitated a more streamlined and coherent analysis.

Furthermore, all enterprises not utilising grid electricity were categorised as operating off-grid. However, a subsequent cross-check based on the location of the enterprise was conducted to assess potential grid access. The dataset provided by Africa Grid [105] was utilised to compare each enterprise location, as obtained from the web-based questionnaire, with the presence of a potentially existing grid connection in that area. Additionally, individual consideration was given to the ISIC category, business activity, value chain, final product(s), and energy carriers of each enterprise. Even in cases where grid connections were potentially available in specific locations, the cross-check process further strengthened the assumption that enterprises without a demand for grid electricity operate off-grid (see Table 6 for more details).

For a comprehensive review of the evaluation results, please refer to Appendix B, Table B2. This Table showcases the outcomes of each enterprise derived from the web-based questionnaire post-data-cleaning, encompassing details such as country, gender-based ownership structure, enterprise size, ISIC category, monthly production output, energy carriers used, type of energy access (on-grid or off-grid), monthly electric, mechanical, and thermal demand levels, and monthly energy expenditure per production output.

### 5.2 Survey limitations

While this study provides valuable insights, several limitations should be acknowledged: Firstly, the relatively limited sample size of 65 respondents across seven African countries is not statistically representative at national or sectoral levels, and hence targets to provide rather analytical than statistical results. It is worth noting that a couple of studies in this field contend with comparable sample size limitations [15,18,46,86]. Additionally, the uneven distribution of the sample across countries might restrict the applicability of the findings to contexts other than those specifically examined. The overrepresentation of Nigeria and varying contributions from other countries may not accurately reflect the broader landscape of enterprises owned by women and men in different regions. The reliance on self-reported data introduces the possibility of recall bias and subjective interpretation, impacting the accuracy of responses. Participants may not accurately remember or report details about the use of energy carriers, expenditures, and related factors, leading to potential inaccuracies. Additionally, the cross-sectional design of the study offers a snapshot of the situation at a specific point in time, limiting the ability to observe changes or trends over an extended period. Furthermore, the exclusion of medium-sized enterprises in the sample restricts the scope, potentially overlooking valuable nuances in larger businesses. Lastly, the dependence on external entities for the outreach campaign may introduce selection bias, influencing the types of enterprises included in the study, and potentially overlooking enterprises that are less accessible through these channels. These factors collectively underscore the need for caution in generalising findings beyond the specific contexts covered in this study.

### 5.3 Findings: a descriptive analysis of productive energy use of enterprises with female participation in ownership across focus countries

This section delves into the findings obtained through a descriptive analysis of the productive use of energy among enterprises with female participation in ownership across the seven focus countries.^8^ Within this section, an overview of various aspects of these enterprises, including their business activities, ISIC categories, and legal statuses is provided. This is followed by assessing productive use patterns, such as type of energy access (on-grid or off-grid), type and number of energy carriers used, level of monthly electric, mechanical, and thermal demand, and corresponding monthly energy expenditures. These variables are compared across the different focus countries, selected industry sectors and distinct gender-based ownership structures of the enterprises (i.e., sole female, female-female, and female-male partnerships).

#### 5.3.1 Business profiles and legal statuses of women-owned enterprises

Following the data cleaning process, a total of 65 valid responses underwent evaluation, with a predominant origin from Nigeria, constituting 40 % of the dataset. Subsequently, contributions from Kenya, Malawi, and Tunisia account for 19 %, 15 %, and 11 %, respectively, while Ghana and Egypt show lower participation rates of nearly 5 % and 6 %, respectively.

A significant majority, comprising almost 79 % of all enterprises, engage in activities within the food sector as per ISIC classifications, including crop and animal production, fishing and aquaculture, as well as the manufacture of food products and food and beverage service activities. The remaining enterprises operate in the textile sector, encompassing the manufacture of textiles, wearing apparel, and leather and related products.

The sample shows an almost equal representation of the three different gender-based ownership structures, with 31 % solely owned by a female, another 31 % adopting a female-female co-ownership model, and 38 % representing female-male co-owned enterprises. Enterprises with a higher proportion of male owners demonstrate a reduced involvement in the textile industry compared to enterprises owned by a sole female or co-owned by female-female partnerships. Specifically, 65–70 % of sole female and female-female enterprises engage in the food sector, while 30–35 % of these enterprises are active in the textile industry. In contrast, 96 % of female-male co-owned enterprises operate within the food sector, with only 4 % venturing into the textile industry. In other words, and when each industry sector is analysed separately, as indicated in Table 4, 53 % of enterprises within the food sector are either solely owned by a female or have a female-female co-ownership structure. Notably, 93 % of textile enterprises are owned by females only (combining sole female-owned and female-female co-owned enterprises).

**Table 4:** Distribution of enterprises by industry sector and gender-based ownership structure.

industry sector	gender-based ownership structure			total
	female only	female-female	female-male	
food sector	27.45 %	25.49 %	47.06 %	100 %
textile sector	42.86 %	50.00 %	7.14 %	100 %

Additionally, variations in business activities can be observed when analysing different gender-based ownership structures within the food and textile sectors: Enterprises with a male included in the ownership structure predominantly engage in relatively similar activities, with a significant proportion focusing on two primary activities: manufacturing of food products (64 %) and crop and animal production (24 %). Conversely, enterprises exclusively owned by women, whether individually or in female-female partnerships, exhibit greater diversity in their business activities. Sole female-owned businesses are primarily engaged in the manufacture of food products (50 %), followed by the manufacture of wearing apparel (25 %), and fishing and aquaculture (10 %). Similarly, enterprises with female-female ownership display a diversified portfolio, with the majority involved in the manufacture of food products (35 %), followed by the manufacture of wearing apparel (25 %), crop and animal production (15 %), and food and beverage service activities (10 %).

Concerning enterprise size based on employee numbers, all respondents fall within the micro category (65 %), or the small category (35 %), aligning with respective national definitions.

Table 5 provides an overview of the respondent distributions categorised by country, industry sector, gender-based ownership structure, and enterprise size.

**Table 5:** Overview of the dataset including number and share of respondents by country, industry sector, gender-based ownership structure, and enterprise size.

country	respondents		industry sector				gender-based ownership structure						enterprise size			
			food		textile		sole female		female -female		female - male		micro		small	
	#	%	#	%	#	%	#	%	#	%	#	%	#	%	#	%
Egypt	4	6.15	2	3.92	2	14.92	4	20.00	0	0.00	0	0.00	3	7.00	1	4.00
Ghana	3	4.62	1	1.96	2	14.92	2	10.00	1	5.00	0	0.00	1	2.00	2	9.00
Kenya	12	18.46	12	23.53	0	0.00	4	20.00	3	15.00	5	20.00	8	19.00	4	17.00
Malawi	10	15.38	6	11.76	4	28.57	2	10.00	6	30.00	2	8.00	6	14.00	4	17.00
Nigeria	26	40.00	23	45.10	3	21.43	5	25.00	6	30.00	15	60.00	19	45.00	7	30.00
Tanzania	3	4.62	0	0.00	3	21.43	0	0.00	2	10.00	1	4.00	0	0.00	3	13.00
Tunisia	7	10.77	7	13.73	0	0.00	3	15.00	2	10.00	2	8.00	5	12.00	2	9.00
**Total**	**65**	**100**	**51**	**78.46**	**14**	**21.54**	**20**	**30.77**	**20**	**30.77**	**25**	**38.46**	**42**	**64.62**	**23**	**35.38**

**Table 6:** Potential off-grid operating enterprises with female participation in ownership by country, location, energy carriers used, ISIC category, business activity, gender-based ownership structure, and grid availability.

# of enterprise	country	location	energy carrier	ISIC category	business activity	gender-based ownership structure	existing grid line
1	Kenya	Mashuru, Kajiado County	gasoline, solar PV, LPG	1	livestock, breeding	female-male	no
2	Kenya	Ruiri, Meru County	solar PV	1	vegetable crops	sole female	yes
3	Kenya	Nairobi, Nairobi City County	solar PV, diesel	1	vegetable crops	female-male	yes
4	Malawi	Lilongwe, Central Region	other	10	flour milling	female-male	yes
5	Nigeria	Abeokuta, Ogun State	LPG, other	3	fish drying	female-female	yes
6	Nigeria	Ijebu Ode, Ogun State	gasoline, LPG	3	fishing, rearing	female-male	yes
7	Nigeria	Ibogun Olaogun, Ogun State	diesel	10	palm oil production	female-male	yes
8	Nigeria	Lafia, Nasarawa State	diesel, other	10	rice milling	female-male	yes
9	Nigeria	Abeokuta, Ogun State	diesel, solar PV, other	10	palm oil production	female-male	yes
10	Nigeria	Ogbomosho, Oyo State	diesel	10	rice milling	female-female	no
11	Nigeria	Ogun State	diesel	10	rice milling	female-male	partially
12	Nigeria	Ota, Ogun State	biomass, other	3	fish drying	sole female	yes
13	Nigeria	Borno State	solar PV	14	tailoring, weaving	sole female	partially
14	Nigeria	Yola North, Adamawa State	biomass	10	flour milling, fish drying	female-female	yes
15	Tunisia	Kebili, Nefzaoua region	solar PV, other	1	plant crops	female-female	no
16	Tunisia	Kebili, Nefzaoua region	other	1	dates, palm trees	female-male	no

Exploring the legal enterprise status within the sample indicates that nearly 62 % of the enterprises are registered, while less than 11 % indicated not being formally registered, and the remaining participants chose to either withhold their response or preferred not to answer. A relatively similar pattern can be observed when exploring the distinct sectors separately: Approximately 61 % of enterprises categorised under the food sector are registered, with 64 % of textile businesses exhibiting a formal registration. Conversely, nearly 10 % and 14 % of enterprises in the food and textile sectors respectively lack registration. The rate of enterprises choosing not to disclose or not to provide a response is substantial, reaching almost 30 % in the food sector and 21 % in the textile sector. Further dissecting the data by gender-based ownership structure, it can be observed that enterprises with sole female ownership lean more towards informal sector status than more gender-diverse enterprises: Among sole female-owned enterprises, 30 % are registered, 10 % are not registered, and 60 % prefer not to disclose or did not respond. Enterprises with a female-female co-ownership structure demonstrate a higher registration rate of 65 %, with 15 % not formally registered, and 20 % preferring not to disclose. This pattern becomes more pronounced among enterprises co-owned by females and males, with 72 % being registered, only 8 % not being formally registered, and 20 % preferring not to disclose or provide a response.

In this sample, sole female-owned and female-female co-owned businesses show a higher inclination towards having a micro-sized enterprise, accounting for 75 % and 70 %, respectively, whereas female-male co-owned enterprises demonstrate a more even distribution between micro- and small-sized enterprises (52 % and 48 %, respectively). This suggests a trend indicating that increased gender diversity in ownership correlates with larger enterprise sizes. To illustrate, among micro-sized enterprises in the study sample, 36 % are solely owned by a female, 33 % by female-female partnerships, and 31 % by female-male co-owned enterprises. This distinction becomes more evident in the distribution among small-sized enterprises: 21 % are solely owned by a female, 26 % by female-female partnerships, and the majority, comprising 52 %, are owned by female-male co-owned enterprises.

#### 5.3.2 Energy use patterns of women-owned enterprises

Analysing the energy use patterns by industry sectors and gender-based ownership structure provides a more nuanced understanding of the diverse productive use dynamics within this dataset.

From the overall sample, it is evident that 25 % of enterprises do not utilise grid electricity and are presumed to function off-grid. This observation becomes particularly interesting when compared with the latest regional and national electrification rates from2021^9^ [106]. In Sub-Saharan Africa, 19 % of urban areas lack grid access, whereas this figure rises to 70 % in rural areas. Conversely, in the Middle East and North Africa region, both urban and rural electrification rates hover slightly below 100 %. Analysing each country in the study sample individually, it becomes apparent that most off-grid operating enterprises originate from Nigeria (62.5 %), followed by Kenya (19 %), Tunisia (12.5 %), and Malawi (6 %), respectively. Notably, in Egypt, Ghana, and Tanzania no off-grid enterprises have been identified in the sample. The latter are also the countries that exhibited relatively low participation rates in the survey, with only 3–4 enterprises from Egypt, Ghana, and Tanzania. Despite Egypt and Tunisia boasting urban electrification rates of 100 % and rural rates of 100 % and 99.7 %, respectively, 2 out of 7 Tunisian enterprises operate off-grid, located in rural areas devoid of grid coverage. Ghana and Tanzania report urban electrification rates of 95 % and 77 %, respectively, with rural rates of 74 % and 24 %. However, all enterprises from these countries in the sample indicated they were on-grid. Malawi exhibits relatively low urban electrification (54 %) and rural electrification rates (less than 6 %), while the sample shows that 1 out of 10 enterprises operates exclusively off-grid. In Kenya, the urban electrification rate is nearly 98 %, with a rural rate of 68 %, yet 3 out of 12 enterprises are working off-grid in the sample. Nigeria demonstrates relatively high urban electrification (89 %) compared to a rural rate of 26 %, with 10 out of 26 enterprises operating off-grid in the sample.

As indicated in Table 6, within the subset of businesses without any grid electricity demand, 62.5 % potentially have access to the grid based on their location and existing grid infrastructure. 12.5 % of enterprises remain uncertain about grid accessibility due to incomplete location data since their locations are only provided at the state/regional level, which partially intersects with grid coverage. The remaining 25 % lack access to any existing grid infrastructure. However, considering factors such as business activity, final production output, and energy carriers used, the assumption was reinforced that all enterprises without a demand for grid electricity operate off-grid. Most of these enterprises are involved in manufacturing food products (44 %), encompassing rice or flour milling and palm oil production, followed by crop and animal production (31 %), including livestock breeding and plantations, as well as fishing and aquaculture (19 %). One enterprise is engaged in the manufacture of wearing apparel (6 %). Predominantly, these enterprises rely on generators fuelled by diesel or petrol, along with solar PV, to fulfil electrical or mechanical needs (75 %). The remaining 25 % cater to thermal demands using LPG, natural gas, kerosene, biomass (i.e., briquettes), and other energy carriers (e.g., firewood and charcoal).

Regarding gender-based ownership structures, 56 % of these off-grid operating enterprises are co-owned by females and males, 25 % are co-owned by females, and 19 % are solely owned by a female. When the sample is stratified by gender-based ownership structure, enterprises with a more gender-diverse ownership structure demonstrate a higher propensity to operate off-grid. Specifically, 36 % of female-male co-owned enterprises operate off-grid, compared to 20 % of female-female co-owned and 15 % of sole female-owned enterprises.

In the overall sample, 43 % of enterprises predominantly use two energy carriers, 35 % rely on a single energy carrier, and 22 % engage with three or more energy carriers. More specifically, 40 % of all enterprises use both grid electricity and fuel, while 25 % rely solely on the grid, and 15 % exclusively use fuel. The remaining enterprises employ a mix of grid electricity, solar PV, and fuel, or rely solely on solar PV.

In the food sector, most enterprises (53 %) use two energy carriers. Meanwhile, 23–24 % rely either on a single energy carrier or embrace a more diversified strategy with three or more carriers. The most prevalent practice within the food sector involves the combination of grid electricity and fuel (47 %), followed by exclusive fuel use (20 %), and exclusive reliance on grid electricity (12 %). The remaining enterprises adopt a multiple energy carrier approach, including a blend of grid electricity, fuel, and solar PV, fuel and solar PV, or a combination of grid electricity and solar PV, as well as solar PV-only. Conversely, the textile sector showcases a distinct energy carrier use trend, primarily leaning towards a singular energy carrier (79 %), while 14 % incorporate three carriers, and only 7 % use two energy carriers. Exclusive reliance on grid electricity emerges as the predominant practice within the textile sector (71 %), while 14 % employ a combination of grid electricity and fuel. The remaining enterprises in this sector use either solar PV-only or a combination of grid electricity and solar PV.

As depicted in Table 7, enterprises solely owned by a female and those with a female-female co-ownership structure exhibit a remarkably similar energy carrier use pattern. Both groups predominantly rely on two energy carriers, followed by one energy carrier, constituting 45 % and 40 %, respectively. Multiple energy carrier use, involving three or four energy carriers, is a less common practice among these enterprises, accounting for only 15 %. It must be noted that they are also engaged in comparable industry sectors, as has been shown in Table 4. In contrast, female-male co-owned enterprises show a distinct trend, with 40 % utilising two carriers (primarily from Nigerian and Kenyan food manufacturing enterprises), 32 % using three or more energy carriers (mainly from Nigerian food manufacturing enterprises), and 28 % relying on a single energy carrier. The relatively high share of female-male co-owned enterprises utilising three or more energy carriers for productive use in the food sector is more than twice as high as for sole female-owned and female-female co-owned enterprises. In other words, 50 % of all enterprises using three or more carriers are owned by enterprises with a female-male co-ownership structure.

**Table 7:** The number of energy carriers utilised in per cent by country and gender-based ownership structure. The Table displays percentages within and across gender-based ownership categories across countries. Columns sum to 100 % within each category. Rows indicating totals within each category sum to 100 %, while the overall sample percentage across gender-based ownership structures sums to 100 %.

country	sole female			female – female			female – male		
# energy carriers	1	2	≥3	1	2	≥3	1	2	≥3
percentage	%	%	%	%	%	%	%	%	%
Egypt	25.00	22.22	0.00	0.00	0.00	0.00	0.00	0.00	0.00
Ghana	0.00	11.11	33.33	0.00	0.00	33.33	0.00	0.00	0.00
Kenya	25.00	11.11	33.33	0.00	22.22	33.33	14.29	20.00	25.00
Malawi	12.50	11.11	0.00	50.00	22.22	0.00	14.29	0.00	12.50
Nigeria	25.00	22.22	33.33	25.00	33.33	33.33	28.57	80.00	62.50
Tanzania	0.00	0.00	0.00	25.00	0.00	0.00	14.29	0.00	0.00
Tunisia	12.50	22.22	0.00	0.00	22.22	0.00	28.57	0.00	0.00
**% within gender-based category**	**40.00**	**45.00**	**15.00**	**40.00**	**45.00**	**15.00**	**28.00**	**40.00**	**32.00**
**% of the total sample**	**12.31**	**13.85**	**4.62**	**12.31**	**13.85**	**4.62**	**10.77**	**15.38**	**12.31**

Sole female-owned enterprises exhibit a notable inclination towards a combination of grid electricity and fuel use (55 %). More diverse enterprises (in terms of both, the number and gender of owners) show a similar pattern where between 30 and 36 % of these enterprises rely on a blend of grid electricity and fuel. The highest share of sole grid electricity reliance is observed among female-female co-owned enterprises (35 %), followed closely by businesses owned by a female (30 %), while only 12 % of female-male enterprises solely rely on the grid to cover electric productive processes. Exclusive fuel use is seen in 24 % and 15 % of female-male and female-female co-owned enterprises respectively, while only 5 % of sole female-owned enterprises solely rely on fuel as an energy carrier. Solar PV (either exclusively or in combination with other carriers) is used by 28 % and 20 % of female-male and female-female co-owned enterprises respectively, and to a lesser extent by sole female-owned businesses (10 %).

While acknowledging the varying energy carrier use in the food and textile sectors, delving into the food sector exclusively offers deeper insights into how enterprise ownership diversity in terms of the number and gender of owners influences energy carrier use. This focus is prompted by the uneven distribution of gender-based ownership structures across industry sectors, particularly the prevalence of sole female-owned and female-female co-owned businesses in the textile sector, which primarily rely on grid electricity. Yet, in alignment with the trends observed across both industry sectors, the food sector reveals a correlation between increased gender diversity in ownership and a greater reliance on fuels and solar PV. Specifically, 25 % and 23 % of female-male and female-female co-owned enterprises respectively, rely exclusively on fuel while this decreases to 7 % among sole female-owned enterprises. Solar PV use, either exclusively or in combination with grid electricity or fuel, is adopted by 29 % and 23 % of female-male and female-female co-owned enterprises respectively, and only by a modest 2 % of sole female-owned businesses. Also consistent with findings from the analysis of both sectors together, most enterprises solely owned by a female use a combination of grid electricity and fuel (71 %), decreasing to 38 % for both female-female and female-male co-owned enterprises.

Among enterprises utilising one or multiple fuels (including diesel, petrol, LPG, kerosene, biomass, and natural gas), constituting 69 % of the overall sample, diesel emerges as the predominant fuel used by 42 % of those enterprises. Following closely, biomass, primarily indicated as firewood, charcoal, or briquettes, accounts for 38 %, while LPG follows at 27 %. Furthermore, 13 % use petrol for productive use, while 11 % use natural gas (stemming from enterprises based in Egypt and Tunisia only), whereby kerosene is utilised by only one enterprise from Kenya.

When examining fuel use among different gender-based ownership structures, distinct patterns emerge: Sole female-owned enterprises mostly use LPG as fuel (31 %), closely followed by natural gas (25 %), and biomass (19 %), with a minority using diesel, petrol, and kerosene respectively. In contrast, enterprises with a female-female co-ownership structure show an equal reliance on LPG and biomass, each accounting for 33 %, with diesel at 27 %, and a minority using natural gas. Conversely, female-male co-owned enterprises display a different trend, predominantly relying on diesel as a fuel (45 %), followed by biomass (31 %), while petrol is used by 17 %, and LPG is used to a minor extent.

Monthly electric and thermal demand levels, assessed by the industry sector, show that in the overall sample, less than 8 % of enterprises, all within the food sector, do not have any electric or mechanical needs, while approximately 55 % do not have any thermal demand, distributed between 67 % in the food sector and 33 % in the textile sector.

Within the subsample of enterprises having electric and mechanical demands, 15 % are uncertain about their electric consumption levels, with 56 % stemming from the food sector and 44 % from the textile sector. Additionally, 27 % consume between 1 and 100 kW-hours equivalent per month (kWheq/month), predominantly from the food sector (75 %), compared to 25 % from the textile sector. Another 25 % consume between 101 and 200 kWheq/month, with 67 % from the food sector and 33 % from the textile sector, while 33 % consume above 200 kWheq/month, mainly from the food sector (95 %) and a minor 5 % from the textile sector. Contrasting with the subsample of enterprises having thermal needs, 62 % are uncertain about their thermal demand, with 89 % in the food sector and 11 % in the textile sector. Furthermore, 28 % consume between 1 and 400 kWheq/month), and a minority of around 3 % consumes between 401 and 600 kWheq/month, between 1,401–1,600 kWheq/month, as well as above 2,000 kWheq/month, all exclusively from the food sector.

Electricity and mechanical demands are found in 90–95 % of enterprises within each gender-based ownership category (see Table 8). Additionally, as (gender) diversity in ownership rises, the percentage of enterprises unaware of their electric and mechanical consumption level decreases, and total monthly electric and mechanical consumption (above 200 kWheq/month) increases. Specifically, 95 % of sole female-owned enterprises have electric and mechanical demands, among which 26 % are uncertain about the amount consumed, 21 % utilise between 1 and 100 kWheq/month, almost 37 % consume between 101 and 200 kWheq/month, and 16 % surpass 200 kWheq/month. In contrast, 90 % of female-female co-owned enterprises have electric and mechanical demands, with 17 % uncertain about their consumption level. Among them, 39 % use between 1 and 100 kWheq/month, 22 % consume between 101 and 200 kWheq/month, and another 22 % exceed 200 kWheq/month. Conversely, 92 % of female-male co-owned enterprises have electric and mechanical demands, with only 4 % uncertain about their consumption level. Within this category, less than 22 % use between 1 and 100 kWheq/month, while 17 % consume between 101 and 200 kWheq/month and 57 % surpass 200 kWheq/month.

**Table 8:** Electric, mechanical, and thermal demand levels for productive use in per cent categorised by consumption brackets, country, and gender-based ownership structure.

Monthly electric and mechanical demand for productive use in kWheq/month															
country	sole female					female – female					female – male				
**kWh**	none	don't know	1–100	101-200	above 200	none	don't know	1–100	101-200	above 200	none	don't know	1–100	101-200	above 200
**percentage**	%	%	%	%	%	%	%	%	%	%	%	%	%	%	%
Egypt	0.00	40.00	0.00	28.57	0.00	0.00	0.00	0.00	0.00	0.00	0.00	0.00	0.00	0.00	0.00
Ghana	0.00	20.00	25.00	0.00	0.00	0.00	33.33	0.00	0.00	0.00	0.00	0.00	0.00	0.00	0.00
Kenya	0.00	20.00	25.00	0.00	66.67	0.00	0.00	28.57	0.00	25.00	0.00	0.00	20.00	25.00	33.33
Malawi	0.00	0.00	25.00	0.00	33.33	0.00	66.67	42.86	25.00	0.00	50.00	0.00	0.00	25.00	0.00
Nigeria	100.00	0.00	25.00	42.86	0.00	100.00	0.00	14.285	25.00	50.00	0.00	0.00	60.00	50.00	66.67
Tanzania	0.00	0.00	0.00	0.00	0.00	0.00	0.00	0.00	25.00	25.00	0.00	100.00	0.00	0.00	0.00
Tunisia	0.00	20.00	0.00	28.57	0.00	0.00	0.00	14.285	25.00	0.00	50.00	0.00	20.00	0.00	0.00
**% within gender-based category**	**5.00**	**25.00**	**20.00**	**35.00**	**15.00**	**10.00**	**15.00**	**35.00**	**20.00**	**20.00**	**8.00**	**4.00**	**20.00**	**16.00**	**52.00**
**% of the total sample**	**1.54**	**7.69**	**6.15**	**10.77**	**4.62**	**3.08**	**4.62**	**10.77**	**6.15**	**6.15**	**3.08**	**1.54**	**7.69**	**6.15**	**20.00**

**Monthly thermal demand for productive use in kWheq/month**															

**country**	**sole female**					**female – female**					**female – male**				
**kWh**	none	don't know	1–400	401-800	above 800	none	don't know	1–400	401-800	above 800	none	don't know	1–400	401-800	above 800

**percentage**	%	%	%	%	%	%	%	%	%	%	%	%	%	%	%

Egypt	20.00	25.00	0.00	0.00	0.00	0.00	0.00	0.00	0.00	0.00	0.00	0.00	0.00	0.00	0.00
Ghana	0.00	25.00	0.00	0.00	0.00	0.00	33.33	0.00	0.00	0.00	0.00	0.00	0.00	0.00	0.00
Kenya	20.00	0.00	0.00	0.00	0.00	0.00	0.00	50.00	0.00	0.00	13.33	14.285	0.00	100.00	100.00
Malawi	20.00	0.00	0.00	0.00	0.00	54.55	0.00	0.00	0.00	0.00	0.00	28.57	0.00	0.00	0.00
Nigeria	3.00	25.00	0.00	0.00	0.00	27.27	33.33	33.33	0.00	00.00	73.33	42.86	100.00	0.00	0.00
Tanzania	0.00	0.00	0.00	0.00	0.00	18.18	0.00	0.00	0.00	0.00	6.67	0.00	0.00	0.00	0.00
Tunisia	10.00	25.00	0.00	0.00	0.00	0.00	33.33	16.67	0.00	0.00	6.67	14.285	0.00	0.00	0.00
**% within gender-based category**	**50.00**	**40.00**	**5.00**	**5.00**	**5.00**	**55.00**	**15.00**	**30.00**	**0.00**	**0.00**	**60.00**	**28.00**	**4.00**	**4.00**	**4.00**
**% of the total sample**	**15.38**	**12.31**	**3.08**	**1.54**	**1.54**	**16.92**	**4.62**	**9.23**	**0.00**	**0.00**	**23.08**	**10.77**	**1.54**	**1.54**	**1.54**

Thermal demand is found in 40–50 % of enterprises within each gender-based ownership category (see Table 8). Similarly to previous correlations made, the awareness level of thermal demand is lowest among enterprises with a sole female owner (80 % do not know their thermal demand), however, followed by more gender-diverse co-ownership structures (i.e., female-male co-owned enterprises) among which 70 % do not know their demand level. Increased awareness is observed among female-female co-owned enterprises among which 33 % do not know their thermal demand. Monthly thermal energy demand above 800 kWheq/month is only found in two enterprises from Kenya. Specifically, one enterprise, solely owned by a female, consumes between 1,401–1,600 kWheq/month, while another enterprise, female-male co-owned, consumes over 2,000 kWheq/month. Furthermore, among both, sole female-owned and female-male co-owned enterprises, 10 % consume between 1 and 400 kWheq/month. Among female-female co-owned enterprises, the majority (67 %) consume between 1 and 400 kWheq/month.

Assessing monthly energy expenditure (combining electricity and fuel costs) in United States dollars per kg production output (USD/kg/month) reveals the following: A relatively high share of 35 % of enterprises lacks available data on monthly energy expenditure, attributed to either unfamiliarity with the price of energy carriers used or the absence of identified production output values (comprising 74 % from the food sector and 26 % from the textile sector). 49 % of enterprises incur costs less than or equal to 1 USD/kg/month, with a significant majority (94 %) belonging to the food sector and a minor 6 % stemming from the textile sector. For 8 % of enterprises, the expenditure ranges from above 1 USD to 10 USD/kg/month, where 25 % are from the food sector and 75 % from the textile sector. Moreover, 3–5% of enterprises pay either above 10 up to 100 USD/kg/month (exclusive to the food sector) or exceed 100 USD/kg/month (40 % from the food sector and 60 % from the textile sector).

Highlighting the influence of gender-based ownership structure on energy expenditure in USD/kg/month, certain trends emerge in the data: As ownership diversity increases in terms of the number and gender of owners, a notable decline in the proportion of enterprises lacking information on energy expenditure per kg output is observed, coupled with a decrease in energy expenditure. Among sole female-owned enterprises, 45 % lack knowledge about energy expenditure. This value decreases to 35 % and 28 % for enterprises with a female-female and female-male partnership structure, respectively. Regarding energy expenses, 30 % of sole female-owned enterprises pay less than or equal to 1 USD/kg/month, as compared to 50 % of female-female co-owned businesses and 64 % of female-male co-owned enterprises. Among sole female-owned enterprises, 5 % incur expenses ranging from above 1 USD to 10 USD/kg/month, 15 % pay above 10 up to 100 USD/kg/month, and another 5 % exceed 100 USD/kg/month. In enterprises with a female-female co-ownership structure, 10 % pay above 1 up to 10 USD/kg/month, and 5 % pay above 10 USD up to 100 USD/kg/month, while none of these enterprises exceed 100 USD/kg/month. As for female-male co-owned enterprises, only 8 % pay above 1 up to 10 USD/kg/month, with no enterprise exceeding 10 USD/kg/month.

## 6 Discussion: how does gender in enterprise ownership correlate with the productive use of energy?

This paper contributes to a nuanced understanding of the productive use of women-owned enterprises. By delving into gender-based ownership structures, a less explored aspect in existing literature, this research categorises women-owned enterprises into three distinct groups: those (i) owned solely by a female, (ii) co-owned by females, and (iii) with a mixed-gender ownership structure herein referred to as female-male co-owned businesses. The sample shows a near-equal distribution of gender-based ownership structures, each representing approximately one-third of the sample, with female-male enterprises constituting the highest share of 38 %.

In line with established databases and available literature [22], this study affirms that women-owned enterprises are primarily categorised as micro or small, with no instances of representation in the medium-sized category within the sample. Analysing the relationship between enterprise size and gender-based ownership structure reveals a significant pattern: Micro-sized enterprises are primarily led by a sole female or by female-female co-owners. As male participation in ownership increases, there is a corresponding shift from micro to small-sized enterprises, reflecting established trends in the existing literature [22]. Further aligning with the literature [4], the study also demonstrates that enterprises with a sole female owner exhibit a greater inclination towards informal sector status compared to more gender-diverse enterprises.

In terms of business activity, both ownership structures, solely owned by a female, and female-female partnerships, demonstrate engagement in the food and textile sectors and showcase a diverse portfolio of activities within these industry sectors as per the definition of ISIC. In contrast, as male ownership increases in enterprises, these businesses tend to be more inclined to work in the manufacture of food products, followed by crop and animal production, and to some extent fishing and aquaculture. This trend may be attributed to gender roles and labour segregation [17], wherein enterprises owned by men exhibit less involvement in the textile sector but are more prominently represented in agriculture, fishing, and aquaculture (as reflected in the sample) [76]. Findings from Ref. [79], where significant disparities in preferences for specific products emerge, corroborate these gender distinctions. For instance, sewing machines are highly ranked among women but considerably less so among men. However, it is important to note that even [79] underscores the prevalence of both men and women as tailors in Africa's informal markets.

Across the focus countries, enterprises with more gender-diverse ownership structures display a greater inclination towards off-grid operations compared to those predominantly owned by females (sole female and female-female partnerships). Examining off-grid operating enterprises by country reveals a consistent trend in terms of gender-based ownership structures, with female-male co-owned enterprises being predominantly represented in off-grid operations in Kenya, Malawi, Nigeria, and Tunisia (no off-grid enterprises have been recorded in Egypt, Ghana, and Tanzania). Notably, within the total subsample of female-male co-owned enterprises, more than one-third (36 %) operate off-grid. While existing literature does not directly address this specific research scope, insights from this study regarding energy expenditure patterns present a compelling hypothesis. The data indicates that more diverse enterprises, especially those with gender-diverse ownership structures, tend to allocate significantly less expenditure per production output compared to sole female and female-female co-owned enterprises. While it holds that in some locations, the absence of a grid necessitates off-grid operations, for the majority, it can be postulated that choosing to operate off-grid may not solely be due to the unavailability of a grid connection, but rather a strategic decision aimed at cost-saving due to the relatively high cost of grid electricity [77]. This hypothesis is consistent with findings from a McKinsey and Company study [107], which suggests that enhanced diversity within enterprises correlates with improved business performance.

The research findings further underscore the potential significance of the enterprise owner's gender on energy carrier use. Female-owned businesses, including solely female and female-female partnerships, exhibit similar trends, primarily relying on one or two energy carriers, mostly grid electricity and fuel. In contrast, female-male co-owned enterprises tend to embrace a more diversified approach, engaging with multiple energy carriers, such as grid electricity, fuel, and solar PV. Furthermore, it can be highlighted that increased gender diversity in ownership is associated with a shift towards exclusive reliance on fuels and increased utilisation of solar PV, representing a departure from the patterns observed in enterprises entirely owned by women (either sole female or female-female partnerships). However, this trend seems to arise from the widespread use of diesel among Nigerian enterprises, with 74 % of all enterprises using diesel originating from Nigeria. Within the Nigerian subsample using diesel for productive use, female-male co-owned enterprises constitute 79 %. Among these Nigerian female-male co-owned enterprises, 45 % operate off-grid, and 55 % operate on-grid. In addition to this, distinguishing fuel choices within women-owned enterprises across all focus countries reveals that diesel, biomass (charcoal, firewood, briquettes), and LPG are the most prevalent. Female-male co-owned enterprises predominantly rely on diesel, followed by biomass, while female-female partnerships exhibit equal reliance on LPG and biomass, followed by diesel. In contrast, sole female-owned enterprises use primarily LPG.

The study results on gender-specific energy carrier use unveil a relatively nuanced landscape that partially aligns with the existing literature examining energy carrier use disaggregated by the sex of the enterprise owners. Some scholarly studies have highlighted that electricity and diesel use is more prevalent among male entrepreneurs [6,16,17,76], while female entrepreneurs tend to lean towards cooking fuels like charcoal, firewood, and gas [6,17,19,57,76]. While this trend is observed within the study sample, grid electricity as an energy carrier is predominantly found in enterprises with exclusive female ownership (either sole female or female-female co-ownership). Furthermore, limited research briefly addressed the observation that male-owned enterprises diversify their energy carriers more than female-owned enterprises [77], a trend corroborated by the present study findings, particularly among more gender-diverse enterprises.

A notable finding emerging from the assessments conducted across the focus countries is the positive correlation between increased ownership diversity (in terms of both, the number and gender of owners) and increased awareness and consumption of electric, and mechanical energy, particularly when surpassing 200 kWheq/month. However, these observations are only partially valid for thermal energy as both sole female-owned and female-male co-owned businesses show relatively low awareness, while as many as 70 % of female-female co-owned enterprises know their thermal demand and consume below 400 kWheq/month. Additionally, enterprises with relatively high thermal demands exceeding 800 kWheq/month are observed across both sole female and gender-diverse ownership structures. While the literature does not directly confirm a link between diversity in ownership and increased awareness [107], has highlighted the general improvement in business performance associated with diversity. Moreover, existing literature suggests that male-owned enterprises typically have higher electric demand compared to their female counterparts, a trend reflected in the study sample by the prevalence of increased electric and mechanical demand among female-male co-owned enterprises.

Analysing energy expenditure in USD/kg/month unveils significant trends potentially influenced by gender-based ownership structure. As ownership diversity increases (in terms of both, the number and gender of owners), a decline in the proportion of enterprises lacking information on energy expenditure is observed, accompanied by an overall decrease in energy expenditure per production output. Specifically, sole female-owned enterprises may incur costs exceeding 100 USD/kg/month, female-female partnered enterprises pay a maximum of 10–100 USD/kg/month, while female-male co-owned enterprises do not exceed 10 USD/kg/month. These findings suggest that diversity in ownership in terms of both, the number and gender of owners, could potentially lead to more efficient energy management and overall enhanced business performance [107]. Increased ownership diversity implies that an owner is not solely responsible for day-to-day operations but has another individual capable of managing and optimising energy resources, including adopting a diversified and cost-efficient approach to energy carriers with various fuels and presumably cost-effective solar PV. Within the assessed countries, it appears that primary reliance on relatively expensive grid electricity tariffs, combined with potentially high grid connection fees, results in high energy expenditures, particularly among enterprises owned solely by a female. However, for enterprises owned solely by a female, connecting to the grid, and using a fuel commonly available and accessible in the country might be the easiest and most convenient option, especially in cases where the owner operates a micro-enterprise with minimal or no employees for further assistance.

## 7 Conclusions and recommendations

This study addressed critical knowledge gaps regarding the productive energy use and ownership structures within WMSME in the food and textile sectors across seven African countries: Egypt, Ghana, Kenya, Malawi, Nigeria, Tanzania, and Tunisia. By formulating two overarching research questions, employing a combination of secondary and primary data collection techniques, and analysing the data using descriptive tools, the study offered valuable insights into the interplay between gender, ownership structures, and productive energy use within WMSME.

The study aimed to address the underrepresentation of WMSME in energy research, particularly emphasising their potential to derive benefits from renewable energy solutions. This area of research has previously received limited attention [11,16,17,19,60]. The research questions focused on identifying viable cases for evaluating the productive use of energy by WMSME across different African countries and industry sectors. The study employed a multidisciplinary approach, integrating qualitative and quantitative methods. Specifically, the research centred on dissecting these patterns based on gender-based ownership structures of the enterprises (i.e., sole female, female-female, and female-male). Limitations of the research design include a restricted in-depth understanding regarding precise energy and electricity use, as well as cost splits of different energy carriers used for productive activities. The need for additional and complementary research emerges, potentially employing statistical correlation analysis, qualitative interviews, or focus group discussions to gain a more profound insight into this field of study. Furthermore, conducting a descriptive analysis involving MSME solely owned by men in similar sectors and countries could offer additional perspectives on energy use patterns and carriers.

The findings revealed notable correlations between the gender-based ownership structure of the assessed enterprises, energy carriers and services used, type of energy access (on-grid or off-grid), awareness levels regarding consumption levels, as well as energy expenditure per kg production output. As ownership diversity increases, a decline in energy expenditure per production output was observed, suggesting more efficient energy management practices. Moreover, female-male co-owned enterprises exhibited a greater inclination towards off-grid operations, indicating strategic decisions aimed at cost-saving. The study also highlighted differences in energy carrier use based on the gender of the enterprise owner. The particularly high reliance on grid electricity among enterprises owned by a female or co-owned by females suggests potential challenges related to national electricity grids in Africa, including electricity tariffs, connection fees, and overall grid reliability.

By implementing the following recommendations, stakeholders can contribute to fostering a more inclusive and sustainable energy ecosystem, driving socio-economic development while preserving the environment [6,20,27,28,46,57,76,77,80].

• The prevalent lack of awareness among many WMSME regarding both energy consumption and expenditure signals a pressing need for capacity-building and training initiatives. Such initiatives should (i) prioritise enhancing awareness levels, (ii) increase efficient resource management practices, and (iii) explore more environmentally friendly alternatives to diesel and other fossil fuels, promoting sustainability and reducing environmental impact.
• Efforts should be made by private, and public stakeholders to enhance the reliability, affordability, and quality of the provided energy supply, supporting both female and male business activities. Enhanced technology adoption by end-users requires a gender-sensitive approach that considers the specific needs and barriers of entrepreneurs, particularly those of WMSME.
• Policy interventions need to be increasingly gender-mainstreamed and should focus on enhancing the affordability and accessibility of energy products and services for WMSME.
• Increased capital flows to the renewable energy sector, competitive financial markets to drive innovation, and tailored financing options for female and male entrepreneurs need to be further leveraged.

## Data availability

The research data associated with the web-based questionnaire (including raw and processed data) has been deposited into Mendeley Data and can be accessed at [108].

## Ethics and consent declarations

This study did not require approval and review by an ethics committee as the research focused on non-sensitive data regarding business operations and energy use, posing minimal risk to participants. Voluntary participation ensured informed consent, with stringent measures taken to anonymise data while remote data collection minimised direct, potentially biased, interactions. Verbal informed consent was obtained due to the impracticality of obtaining written consent across seven African countries and engaging participants remotely. Participants were assured of anonymity and willingly provided verbal consent, endorsing the publication of their anonymised case details.

## CRediT authorship contribution statement

**Djalila Gad:** Writing – review & editing, Writing – original draft, Visualization, Validation, Resources, Methodology, Investigation, Formal analysis, Data curation. **Pierluigi Leone:** Writing – review & editing, Validation, Supervision, Resources, Methodology, Conceptualization.

## Declaration of competing interest

The authors declare that they have no known competing financial interests or personal relationships that could have appeared to influence the work reported in this paper.

## Figures and Tables

**Table A.1:** Web-based questionnaire form, including type of answer choices, answer choices, notes (if applicable), and rationale for questions posed

Introduction	Thank you for participating in this online survey on energy use in textile and food industries. This survey has a total of two sections and 14 questions which will take approximately 15 min of your time.			Rationale for questions (not visible to research participants)
Section 1	FIRST SECTION: At first, we would like to know more about the enterprise. This section has 10 questions.			
Question	Type of answer choice	Answer choices	Notes (if applicable)	
1.1. Are you the owner of a micro, small or medium-sized enterprise in the textile or food sector?	Multiple Choice	“Yes”, “No“	n/a	Identification of respondent and their role in the enterprise
1.2. In which **country** is the headquarters of the enterprise?	Dropdown (one answer choice possible only)	“Egypt”, “Ghana”, “Kenya”, “Malawi”, “Tanzania”, “Tunisia”	n/a	Geographical location
1.3. In which **city**, **town**, **or village** is the enterprise located?	Short answer	–	If the enterprise has more than one location, please write down the names of all locations.	Urban/rural setting
1.4. Please indicate the **gender of the owner**.	Multiple Choice (one answer choice possible only)	“Female”, “Male”, “Other”, “Prefer not to say”	n/a	Ownership structure
1.5. Please indicate the **gender of the co-owner** (if any).	Multiple Choice (one answer choice possible only)	“Female”, “Male”, “Other”, “Prefer not to say”, “No co-owner”	n/a	
1.6. Please write down the **number of employees** of the enterprise.	Short answer	–	(a) Value doesn't include the owner and co-owner (if any). (b) The value can be equal or above zero.	Enterprise size
1.7. Please indicate whether the enterprise is **registered** and/or **licensed**.	Multiple Choice (one answer choice possible only)	“Yes”, “No”, “I prefer not to say”, “I don't know”	n/a	Formal/legal enterprise status
1.8. Please select the **sector** that describes the enterprise best.	Dropdown (one answer choice possible only)	[Selected ISIC activities under ISIC divisions 01, 02, 03, 10, 11, 12, 13, 14, 15, 16, 31, 32, and 56 which relate to the food and textile sector]	n/a	Business sector identification
1.9. Please describe in your own words, the **activities** of the enterprise.	Paragraph	–	(a) Activities in the textile sector could be for example: production of textiles/garments/hides & skins, i.e., preparing raw material, spinning, knitting, weaving, dyeing, printing, finishing, sewing, etc. (b) Activities in the food sector could be for example: wheat/maize milling, drying fish/vegetables, milk production/storage, livestock breeding, etc.	cross-checking of exact business activity
1.10. Please write down the **annual production output** of the enterprise.	Paragraph	–	(a) Please indicate value, unit, and produced item. The response could be for example: 800 kg tomato purée/year, or 1000 L olive oil/year, or 1500 leather shoes/year. (b) Please list all production outputs.	production output (in combination with question 2.4)

**Section 2**	SECOND SECTION: We would now like to understand the extent to which energy is used in the enterprise. This last section has 4 questions.			

**Question**	**Type of answer choice**	**Answer choices**	**Notes (if applicable)**	
2.1. Please select the **source of energy** that is used in the enterprise for its energetic and electric needs.	Checkboxes (multiple answers choices possible)	“Electricity from the national grid”, “Diesel”, “Gasoline”, “Kerosene”, “Solar energy”, “Biomass”, “LPG”, “Natural gas”, “Other”, “None”, “I don't know”	(a) It is possible to select more than one energy source.	energy carrier use
2.2. Please select the **average monthly electricity consumption** level that reflects best the electric needs of the enterprise.	Dropdown (one answer choice possible only)	[Consumption brackets from 0 to 20 kWh until 181–200 kWh in 20 kWh ranges respectively], “Above 200 kWh”, “None”, “I don't know”	(a) Only select from options, if applicable. Otherwise, select “none". (b) If you don't know the electricity consumption (given in kWh), you can estimate it by multiplying the wattage of a device with the hours per month the device is working, e.g. a light bulb of 100 W working for 30 h per month consumes: 100W × 30 h = 3000 Wh = 3 kWh.	consumption level
2.3. Please indicate the **average monthly fuel consumption** that reflects best the energy needs of the enterprise.	Short answer	–	(a) If not applicable, write “none". (b) Please indicate value, unit, and energy source. The response could be for example: 20 L diesel/month, or 30 kg LPG/month, etc. (c) If you do not know the answer, write “I don't know".	
2.4. Please write down the **yearly** **average energy costs (electricity and fuel)** of the enterprise.	Short answer	–	Notes: (a) Please insert the average cost of 2022 and indicate the value in local currency. (b) If you do not know the answer, write “I don't know".	energy expenditure (in combination with question 1.10)

**Table B.1:** Conversion factors for web-based questionnaire evaluation

Fuel conversion factors to kilowatt-hour equivalent		
Type of fuel (weight or volume)	**Conversion factor**	**equipment efficiency (electric and thermal)** [[87], [88], [89], [90], [91], [92]]
LPG (1 kg)	13.6	57 % (thermal)
petrol (1L)	9.6	30 % (electric)
diesel (1L)	10	30 % (electric)
briquettes (1 kg)	4.9	60 % (thermal)
natural gas (1 kg)	14	60 % (thermal)
charcoal (1 kg)	8.05	22 % (thermal)
firewood (1 kg)	4.1	22 % (thermal)
**Monthly production output conversions** [[93], [94], [95], [96], [97]]		
**Type of production output**	**Weight of production output**	
garments and clothes	0.047kg/piece	
accessories	0.016kg/piece	
pair of shoes	1kg/pair (assumed)	
baked goods	0.25kg/piece (assumed)	
cow	450.83kg/cow	
goat	31.25kg/goat	
chicken	1kg/chicken (assumed)	
dried fish	0.1kg/piece	
meal	0.5kg/meal (assumed)	
packet of maize	1kg/packet (assumed)	
trey of eggs	0.323kg/trey	
**Average exchange rates for the year 2022** [[98], [99], [100], [101], [102], [103], [104]]		
**Local currency**	**Conversion factor for United States dollars (USD)**	
Egyptian pound (EGP)	19.15	
Ghanaian cedi (GHC)	8.45	
Kenyan shilling (KES)	117.83	
Malawian kwacha (MWK)	1,023.80	
Nigerian naira (NGN)	422.45	
Tanzanian shilling (TSh)	2,332.24	
Tunisian dinar (TND)	3.11	

**Table B.2:** Results obtained from the web-based questionnaire after the data cleaning process, including country, gender-based ownership structure, enterprise size, ISIC division, monthly production output, energy carriers used, type of energy access, monthly electric, mechanical, and thermal demand levels, and monthly energy expenditure per production output

#	Country	Gender-based ownership structure	Enterprise size	ISIC division	Monthly production [kg/month]	Energy carriers	energy access type	Monthly electric and mechanical demand [kWheq/month]	Monthly thermal demand [kWheq/month]	Monthly energy expenditure per production output [USD/kg/month]
1	Egypt	sole female	small	13	5.88	GE	on-grid	121–140	none	2.963
2	Egypt	sole female	micro	14	3.92	GE	on-grid	n/a	none	n/a
3	Egypt	sole female	micro	10	21.25	GE, NG	on-grid	n/a	n/a	n/a
4	Egypt	sole female	micro	10	266.67	GE, NG	on-grid	121–140	n/a	0.057
5	Ghana	female-female	small	14	5.04	GE, D, LPG	on-grid	n/a	n/a	21.141
6	Ghana	sole female	micro	10	500.00	GE, LPG	on-grid	81–100	n/a	0.039
7	Ghana	sole female	small	14	3.15	GE, D, LPG	on-grid	n/a	n/a	n/a
8	Kenya	female-male	micro	1	3,505.00	P, S, LPG	off-grid	81–100	604.66	n/a
9	Kenya	sole female	small	10	n/a	GE	on-grid	above 201	none	n/a
10	Kenya	sole female	micro	56	1,608.58	GE, K, LPG, O	on-grid	above 201	201.55	0.018
11	Kenya	female-female	small	56	98,606.00	GE, LPG, O	on-grid	81–100	310.08	0.004
12	Kenya	female-female	micro	10	458.33	GE, LPG	on-grid	81–100	232.56	0.208
13	Kenya	sole female	micro	1	16,250.00	S	off-grid	n/a	none	0.000
14	Kenya	female-male	micro	1	120.00	GE	on-grid	above 201	none	n/a
15	Kenya	female-only	micro	10	750.00	GE, LPG	on-grid	21–40	1,472.88	0.328
16	Kenya	female-male	micro	10	4,000.00	GE, B	on-grid	101–120	n/a	0.053
17	Kenya	female-female	micro	56	376.00	GE, O	on-grid	above 201	80.00	0.036
18	Kenya	female-male	small	10	1,999,592.11	GE, D, S, B	on-grid	above 201	141,120.00	0.001
19	Kenya	female-male	small	1	64,166.67	S, D	off-grid	above 201	none	0.00004
20	Malawi	sole female	micro	14	3.92	GE	on-grid	81–100	none	0.025
21	Malawi	sole female	small	10	1,104.17	GE, D	on-grid	above 201	none	110.576
22	Malawi	female-female	micro	15	12.25	GE	on-grid	181–200	none	n/a
23	Malawi	female-female	small	1	208.33	GE	on-grid	n/a	none	0.008
24	Malawi	female-female	micro	10	208.33	GE	on-grid	n/a	none	n/a
25	Malawi	female-female	micro	1	100.00	GE, S	on-grid	41–60	none	n/a
26	Malawi	female-male	small	10	125,000.00	O	off-grid	none	n/a	0.0001
27	Malawi	female-female	micro	14	97.92	GE, S	on-grid	0–20	none	n/a
28	Malawi	female-male	small	1	1,166.67	GE, S, B, O	on-grid	101–120	n/a	n/a
29	Malawi	female-female	micro	14	3.92	GE	on-grid	0–20	none	n/a
30	Nigeria	female-female	small	10	4,750.00	GE, D, S, LPG	on-grid	41–60	387.60	0.038
31	Nigeria	female-male	small	10	125,000.00	GE, D, B	on-grid	above 201	n/a	0.008
32	Nigeria	female-female	micro	3	250.00	LPG, O	off-grid	none	232.56	0.059
33	Nigeria	female-male	micro	3	500.00	P, LPG	off-grid	141–160	232.56	0.284
34	Nigeria	female-male	micro	10	750.00	D	off-grid	141–160	none	0.097
35	Nigeria	female-male	small	10	32,500.00	D, O	off-grid	above 201	none	0.041
36	Nigeria	female-male	micro	10	116,667.67	GE, D	on-grid	above 201	none	0.008
37	Nigeria	female-male	small	10	41,666,666.67	GE, D	on-grid	above 201	none	0.00001
38	Nigeria	female-female	micro	10	5,500.00	GE, D	on-grid	above 201	none	0.015
39	Nigeria	female-male	small	10	6,666.67	GE, D, S	on-grid	above 201	none	0.030
40	Nigeria	sole female	micro	3	166.67	GE, P	on-grid	121–140	none	n/a
41	Nigeria	female-male	micro	10	416.67	D, S, O	off-grid	21–40	n/a	n/a
42	Nigeria	female-male	micro	3	183.33	GE, P, O	on-grid	above 201	n/a	0.323
43	Nigeria	female-male	micro	10	1,750.00	GE, P	on-grid	81–100	none	0.068
44	Nigeria	female-female	micro	10	30.416.67	D	off-grid	above 201	none	0.012
45	Nigeria	female-male	small	10	16,666.67	GE, D	on-grid	above 201	none	n/a
46	Nigeria	female-male	micro	10	16,666.67	GE, D	on-grid	above 201	none	n/a
47	Nigeria	female-male	micro	10	333.33	GE, S, P	on-grid	21–40	none	0.010
48	Nigeria	female-male	small	10	12,500.00	D	off-grid	above 201	none	0.028
49	Nigeria	female-male	micro	10	4,166.67	GE, D	on-grid	above 201	none	0.014
50	Nigeria	sole female	micro	10	n/a	GE, LPG, O	on-grid	161–180	n/a	n/a
51	Nigeria	female-female	micro	14	3.92	GE	on-grid	101–120	none	0.252
52	Nigeria	sole female	micro	3	833.33	B, O	off-grid	none	n/a	n/a
53	Nigeria	sole female	micro	14	3.92	GE	on-grid	21–40	none	10.075
54	Nigeria	sole female	micro	14	1.96	S	off-grid	141–160	none	n/a
55	Nigeria	female-female	micro	10	151.67	B	off-grid	none	n/a	n/a
56	Tanzania	female-female	micro	14	4.70	GE	on-grid	above 201	none	9.123
57	Tanzania	female-male	micro	14	3.92	GE	on-grid	n/a	none	8.758
58	Tanzania	female-female	micro	13	23.50	GE	on-grid	181–200	none	3.649
59	Tunisia	female-female	micro	10	10,833.33	GE, NG	on-grid	141–160	168.00	0.025
60	Tunisia	sole female	micro	10	10,833.33	GE, NG	on-grid	161–180	n/a	14.865
61	Tunisia	sole female	micro	10	7,083.33	GE	on-grid	n/a	none	n/a
62	Tunisia	sole female	micro	10	10,833.33	GE, NG	on-grid	161–180	n/a	n/a
63	Tunisia	female-female	micro	1	n/a	S, O	off-grid	0–20	n/a	n/a
64	Tunisia	female-male	micro	1	333.33	GE	on-grid	41–60	none	n/a
65	Tunisia	female-male	micro	1	89.17	O	off-grid	none	n/a	n/a

## Appendix B

where B = biomass; D = diesel; GE = grid electricity; K = kerosene; LPG = liquified petroleum gas; NG = natural gas; O = other; P = petrol; S = solar PV.

## Footnotes

2 Throughout the paper, the term “on-grid” has been consistently chosen, aligning with usage in peer-reviewed publications of similar research fields [13,109]. This term is synonymous with “grid electricity”, “grid-connected”, and “grid-tied”, and contrasts with the term “off-grid".

## Footnotes

1 The term “productive use of energy” is interchangeable with the terms “productive energy use”, “productive engagements”, and “productive activities”.

3 The term energy carrier is employed throughout this paper to indicate end-use energy transformed from a primary energy source.

4 For more detailed information on the Environmental Kuznets Curve (EKC) hypothesis, kindly refer to the specified sources indicated by Refs. [27,28], as elaborating further on this topic would extend beyond the scope of this research.

5 The focus on electricity in the literature concerning productive energy use stems from its prevalence as the primary energy carrier in such discussions [5].

6 The World Bank introduces the concept of “Modern Energy Cooking Services (MECS)" within the framework of the World Bank's Multi-tier Framework (MTF). MECS considers various factors affecting cooking, such as exposure, efficiency, convenience, safety, affordability, and fuel availability [63].

7 While the web-based questionnaire exclusively offered solar energy as a selectable response, it is inferred that enterprises indicating the use of solar energy employ solar photovoltaic (PV) technology.

8 The dataset (including raw and processed data) can be accessed at [108].

9 It is essential to note that electrification rates may not directly correlate with the on-grid and off-grid operations of enterprises within the sample. National electrification rates typically encompass the entire population, whereas the sample focuses specifically on productive energy use within enterprises. Albeit certain limitations, national electrification rates can offer valuable insights and perspectives regarding the broader context of energy access.

## References

[1] Ojong N., Simba A., Dana L.-P. (2021). Female entrepreneurship in Africa: A review, trends, and future research directions. J. Bus. Res..

[2] Jaiyeola E.F., Adeyeye M.M. (2021). Obstacles along the path of women enterprises in Africa: A case study of Ogotun women in Ekiti state, Nigeria. Heliyon.

[3] Ferroukhi R., Renner M., Nagpal D., García-Baños C., Barua B., Renewable energy: A gender perspective, 2019 Abu Dhabi https://www.irena.org/-/media/Files/IRENA/Agency/Publication/2019/Jan/IRENA_Gender_perspective_2019.pdf?rev=bed1c40882e54e4da21002e3e1939e3d. (Accessed 25 December 2023).

[4] Rojas A.V., Schmitt F.M., Aguilar L., Guidelines on renewable energy technologies for women in rural and informalurban areas, 2012. https://portals.iucn.org/union/sites/union/files/doc/guidelines_on_renewable_energy_technologies_for_women_in_rural_and_informal_urban_areas.pdf. (Accessed 25 December 2023).

[5] Pueyo A., Maestre M. (2019). Linking energy access, gender and poverty: A review of the literature on productive uses of energy. Energy Res. Social Sci..

[6] Ngoo G., Kooijman A., Gender and energy country briefs: Tanzania, 2020. https://www.energia.org/assets/2021/02/Country-brief-Tanzania_Nov2020_final.pdf. (Accessed 4 May 2024).

[7] Fridriksson T., Schomer I., Janik V.L., Gender equality in the geothermal energy sector, Road to sustainability, 2019. https://documents1.worldbank.org/curated/en/678101556890345718/pdf/Gender-Equality-in-The-Geothermal-Energy-Sector-Road-to-Sustainability.pdf. (Accessed 4 May 2024).

[8] Schiffer A., Yesutanbul A.N, Energy access and gender in Ghana, Policy brief, Cambridge, 2021. https://genderenergycompact.org/document/energy-access-and-gender-in-ghana-policy-brief/. (Accessed 4 May 2024).

[9] Ouziaux S., Mouffe M., Fragkos P., Chaumont S., Naffah E., Melgar F., Demkova D., Charalampidis I., ASSET study: Collection of gender-disaggregated data on the employment and participation of women and men in the energy sector, 2021. https://op.europa.eu/en/publication-detail/-/publication/2c7e5b81-15cd-11ec-b4fe-01aa75ed71a1/language-en. (Accessed 4 May 2024).

[10] Muza O., Thomas V.M. (2022). Cultural norms to support gender equity in energy development: Grounding the productive use agenda in Rwanda. Energy Res. Social Sci..

[11] Patnaik S., Jha S., Jain T., Improving women’s productivity and incomes through clean energy in India, 2021. https://www.ceew.in/publications/improving-womens-productivity-through-clean-energy. (Accessed 4 May 2024).

[12] Kothari C.B., Methodology research (2004).

[13] Pradhan Shrestha R., Jirakiattikul S., Lohani S.P., Shrestha M. (2023). Perceived impact of electricity on productive end use and its reality: Transition from electricity to income for rural Nepalese women. Energy Pol..

[14] Ingole C.K. (2023). Sustainability of productive use of off-grid renewable energy: A case of a women’s collective from rural India. Int. J. Manag. Sustain..

[15] Ceschin F., Petrulaityte A., Musango J.K., Mwiti B.K. (2023). Mainstreaming gender in energy design practice: Insights from companies operating in sub-saharan Africa’s energy sector. Energy Res. Social Sci..

[16] Asibey M.O., Ocloo K.A., Amponsah O. (2021). Gender differences and productive use of energy fuel in Ghana's rural non-farm economy. Energy.

[17] Pueyo A., Bawakyillenuo S., Carreras M. (2020). Energy use and enterprise performance in Ghana: How does gender matter?. Eur. J. Dev. Res..

[18] Wiese K. (2020). Energy 4 all? Investigating gendered energy justice implications of community-based micro-hydropower cooperatives in Ethiopia. Innovat. Eur. J. Soc. Sci. Res..

[19] Pueyo A., Carreras M., Ngoo G. (2020). Exploring the linkages between energy, gender, and enterprise: Evidence from Tanzania. World Dev..

[20] Ferreri J., Singh N., Schomer I., Zwank D. (2022). Building the business case for women's inclusive financing in last-mile renewable energy markets in sub-saharan Africa. https://energia.org/document/building-the-business-case-for-womens-inclusive-financing-in-last-mile-renewable-energy-markets/.

[21] Das I., Klug T., Krishnapriya P.P., Plutshack V., Saparapa R., Scott S., Sills E., Jeuland M., Kara N., Pattanayak S. (2020. https://energyaccess.duke.edu/publication/a-virtuous-cycle-reviewing-the-evidence-on-womens-empowerment-and-energy-access-frameworks-metrics-and-methods/. (Accessed 4 May 2024). A virtuous cycle? Reviewing the evidence on women’s empowerment and energy access, frameworks. metrics and methods.

[22] SME Finance Forum, MSME economic indicators, 2023. https://www.smefinanceforum.org/data-sites/msme-country-indicators. (Accessed 25 December 2023).

[23] International Energy Agency (IEA), Gender and energy data explorer, 2023. https://www.iea.org/data-and-statistics/data-tools/gender-and-energy-data-explorer. (Accessed 25 December 2023).

[24] The World Bank Group, World Bank enterprise surveys (WBES), 2023. https://www.enterprisesurveys.org/en/enterprisesurveys. (Accessed 25 December 2023).

[25] Regmi P.R., Waithaka E., Paudyal A., Simkhada P., Van Teijlingen E. (2016). Guide to the design and application of online questionnaire surveys. Nepal J Epidemiol.

[26] Andrews D., Nonnecke B., Preece J., Conducting research on the internet: Online survey design, development and implementation guidelines, Int. J. Hum. Comput. Interact. 16 (2003) 185–210. https://www.researchgate.net/publication/228597952_Conducting_Research_on_the_Internet_Online_Survey_Design_Development_and_Implementation_Guidelines. (Accessed 1 April 2024).

[27] Işık C., Simionescu M., Ongan S., Radulescu M., Yousaf Z., Rehman A., Alvarado R., Ahmad M. (2023). Renewable energy, economic freedom and economic policy uncertainty: New evidence from a dynamic panel threshold analysis for the G-7 and BRIC countries. Stoch. Environ. Res. Risk Assess..

[28] Işık C., Bulut U., Ongan S., Islam H., Irfan M. (2024). Exploring how economic growth, renewable energy, internet usage, and mineral rents influence CO2 emissions: A panel quantile regression analysis for 27 OECD countries. Resour. Pol..

[29] Pollitzer E., Lee H., Applying gender lenses to the interlinkages and synergies between SDGs, Making sure that agenda 2030 will not leave women behind, 2020. https://www.globalwomennet.org/wp-content/uploads/2021/02/Applying_gender_lens_to_the_interlinkages_and_synergies_betweenSDGs.pdf. (Accessed 4 May 2024).

[30] Işık C., Ongan S., Islam H. (2024). A new pathway to sustainability: Integrating economic dimension (ECON) into ESG factors as (ECON-ESG) and aligned with sustainable development goals (SDGs). Journal of Ekonomi.

[31] Işık C., Ongan S., Islam H., Jabeen G., Pinzon S. (2024). Is economic growth in East Asia Pacific and South Asia ESG factors based and aligned growth?. Sustain. Dev..

[32] Işık C., Ongan S., Islam H., Pinzon S., Jabeen G. (2024). Navigating sustainability: Unveiling the interconnected dynamics of ESG factors and SDGs in BRICS‐11. Sustain. Dev..

[33] Aarakit S.M., Ntayi J.M., Wasswa F., Buyinza F., Adaramola M.S. (2024). Conceptualization and antecedents of productive use of electricity: A systematic literature review. Clean Eng Technol.

[34] Cabraal R.A., Barnes D.F., Agarwal S.G. (2005). Productive uses of energy for rural development. Annu. Rev. Environ. Resour..

[35] Kapadia K., Productive uses of renewable energy: A review of four bank-GEF projects. 2004. http://www.martinot.info/Kapadia_WB.pdf. (Accessed 4 January 2024).

[36] Bhatia M., Angelou N., Beyond connections: Energy access redefined, ESMAP Technical Report 008/15, 2015. https://openknowledge.worldbank.org/handle/10986/24368. (Accessed 2 February 2024).

[37] Heidari S., Babor T.F., De Castro P., Tort S., Curno M. (2016). Sex and gender equity in research: Rationale for the SAGER guidelines and recommended use. Res Integr Peer Rev.

[38] Van Epps H., Astudillo O., Del Pozo Martín Y., Marsh J. (2022). The sex and gender equity in research (SAGER) guidelines: Implementation and checklist development. Eur. Sci. Ed..

[39] Dutta S., Kooijman A., Cecelski E., Energy access and gender, Getting the right balance, 2017. https://documents1.worldbank.org/curated/en/463071494925985630/pdf/115066-BRI-P148200-PUBLIC-FINALSEARSFGenderweb.pdf. (Accessed 4 May 2024).

[40] Edomah N., Foulds C., Malo I., Energy access and gender in Nigeria, Policy brief, 2021. https://energia.org/document/energy-access-and-gender-in-nigeria-policy-brief/#:~:text=Over%2085%20million%20Nigerians%20(amounting,13.5%25%20for%20women%20than%20men. (Accessed 4 May 2024).

[41] De Groot J., Mohlakoana N., Knox A., Bressers H. (2017). Fuelling women’s empowerment? An exploration of the linkages between gender, entrepreneurship and access to energy in the informal food sector. Energy Res. Social Sci..

[42] Ngum S., Kim L., Power a gender-just energy transition, 2023. https://www.greenpolicyplatform.org/sites/default/files/downloads/resource/FINAL_230315_GGKP_Gender_Report%5B13%5D_0.pdf. (Accessed 4 May 2024).

[43] García-Baños C., Renner M., Solar PV: A gender perspective, 2022. https://www.irena.org/Publications/2022/Sep/Solar-PV-Gender-Perspective. (Accessed 4 May 2024).

[44] Acuna N., Sans N.J.M., Hallgrimsdottir E., Berkhouch Y., Power with full force, Getting to gender equality in the hydropower sector, 2023. https://www.esmap.org/Gender-and-Hydropower. (Accessed 4 May 2024).

[45] Ferroukhi R., Renner M., García-Baños C., Wind energy: A gender perspective, 2020. https://www.irena.org/Publications/2020/Jan/Wind-energy-A-gender-perspective. (Accessed 4 May 2024).

[46] Jreich R., A just transition or just a transition: Making the case for women in energy, 2024. https://res4africa.org/news/2024/a-just-transition-or-just-a-transition-making-the-case-for-women-in-energy/. (Accessed 4 May 2024).

[47] Boyd A., Nobelius A.-M., Stands S., Women for sustainable energy, Strategies to foster women’s talent for transformational change, 2019. https://www.globalwomennet.org/women-for-sustainable-energy/. (Accessed 4 May 2024).

[48] Cakmak Z., Celik O., Ucar R.C., Budak S., Hatipoglu S., Yildiz D., Budak I., Gender equality in energy industry report, 2022. https://www.globalwomennet.org/gender-equality-in-energy-industry-report/. (Accessed 4 May 2024).

[49] Kraft C., Qayum S., Pröstler K., Schuber C., Gender equality in the sustainable energy transition, 2023. https://www.unwomen.org/sites/default/files/2023-05/Gender-equality-in-the-sustainable-energy-transition-en.pdf. (Accessed 4 May 2024).

[50] Orlando M.B., Janik V.L., Vaidya P., Angelou N., Zumbyte I., Adams N., Getting to gender equality in energy infrastructure, Lessons from electricity generation, transmission, and distribution projects, 2018. (Accessed 4 May 2024).

[51] Asian Development Bank (ADB), Accelerating gender equality in the renewable energy sector, 2022. https://www.adb.org/publications/gender-equality-renewable-energy-sector. (Accessed 4 May 2024).

[52] Chase J., Hayim L., Glatthaar A., Ferroukhi R., Khalid A., García-Baños C., Women in clean energy, Middle East and North Africa survey 2017, 2017. https://data.bloomberglp.com/professional/sites/24/2017/05/2017-05-03-BNEF-CEBC-IRENA-MENA-Women-in-Clean-Energy-Final.pdf. (Accessed 4 May 2024).

[53] Ferroukhi R., Nagpal D., Parajuli B., Policies and regulations for renewable energy mini-grids, 2018. https://www.irena.org/publications/2018/Oct/Policies-and-regulations-for-renewable-energy-mini-grids. (Accessed 4 May 2024).

[54] Asian Development Bank (ADB), Gender tool kit: Energy going beyond the meter, 2012. https://www.adb.org/sites/default/files/institutional-document/33650/files/gender-toolkit-energy.pdf. (Accessed 4 May 2024).

[55] Mohideen R., Energy technology innovation in South Asia, Implications for gender equality and social inclusion, 2018. 10.22617/WPS179175-2. (Accessed 4 May 2024).

[56] Ferroukhi R., Nagpal D., Parajuli B., Alexander S., Fostering livelihoods with decentralised renewable energy, An ecosystems approach, 2022. https://www.irena.org/publications/2022/Jan/Fostering-Livelihoods-with-Decentralised-Renewable-Energy. (Accessed 4 May 2024).

[57] Clancy J., Dutta S., Women and productive uses of energy: Some light on a shadowy area, 2005. https://www.energia.org/assets/2015/06/43-Women-and-productive-use-of-energy.pdf. (Accessed 4 May 2024).

[58] Practical Action, Mainstreaming gender in national energy policy and plans, Learning from Kenya’s journey and success, 2023. https://practicalaction.org/knowledge-centre/resources/mainstreaming-gender-in-national-energy-policy-and-plans-learning-from-kenyas-journey-and-success/. (Accessed 4 May 2024).

[59] André K., Lehner B., Ngei D., Lins C., Furuya S., Law S., McCue O., Oliphant M., Skowron A., Gsänger S., Tai K., Weckend S., Bhagirath A., Oualid R., Viskantaite G., Ferroukhi R., Community energy toolkit: Best practices for broadening the ownership of renewables, 2021. https://www.irena.org/Publications/2021/Nov/Community-Energy-Toolkit-Best-practices-for-broadening-the-ownership-of-renewables. (Accessed 4 May 2024).

[60] ENERGIA, Asian Development Bank (ADB), Japan Fund for Prosperous and Resilient Asia and the Pacific (JFPR), Towards gender equality and social inclusion in energy utilities: Approaches, methods and results from Nepal, 2022. https://www.adb.org/projects/documents/nep-50059-002-dpta. (Accessed 4 May 2024).

[61] United Nations Development Programme (UNDP), Energy and gender for sustainable development: A toolkit and resource guide, 2004. https://www.undp.org/sites/g/files/zskgke326/files/publications/genderengtoolkit.pdf. (Accessed 25 December 2023).

[62] Energy Sector Management Assistance Programme (ESMAP), Building evidence to unlock impact finance, A field assessment of clean cooking co-benefits for climate, health, and gender, 2023. https://documents.worldbank.org/en/publication/documents-reports/documentdetail/099051123130561434/p17423201c1bc105d0a4da0803634916bb0. (Accessed 4 May 2024).

[63] Khanna R., Zhang Y., Durix L., Pinto A., Adongo Ochieng C., Wu J., Wang Y., The state of access to modern energy cooking services, 2020. https://www.worldbank.org/en/topic/energy/publication/the-state-of-access-to-modern-energy-cooking-services. (Accessed 4 May 2024).

[64] Acuña Castillo N., Zhang Y., Adongo Ochieng C., Adams N., Schomer I., Wu J., Pinto A., Greene J., Morris E., Matinga M., Opening opportunities, closing gaps, advancing gender-equal benefits in clean cooking operations, 2022. https://documents.worldbank.org/en/publication/documents-reports/documentdetail/099215003152218466/p1742320beb6090670933705085ff1c047b. (Accessed 4 May 2024).

[65] Berthelemy J.-C., Challenges of decentralized electrification for economic development: Lessons from experience, 2019. https://ferdi.fr/dl/df-F6BTHiMws1iXRmddVTfuSiM6/ferdi-b194-challenges-of-decentralized-electrification-for-economic.pdf. (Accessed 4 May 2024).

[66] Köhlin G., Sills E. O., Pattanayak S. K., Wilfong C., Energy, gender and development, What are the linkages? Where is the evidence?, 2011. https://elibrary.worldbank.org/doi/abs/10.1596/1813-9450-5800#:∼:text=The%20report’s%20main%20finding%20is,preferences%2C%20opportunity%20cost%20of%20time. (Accessed 4 May 2024).

[67] United Nations Environment Programme (UNEP), Powering equality: Women’s entrepreneurship transforming Asia’s energy sector, 2020. https://www.unep.org/resources/report/powering-equality-womens-entrepreneurship-transforming-asias-energy-sector. (Accessed 4 May 2024).

[68] Maier E., Constant S., Ahmad A., Gender in energy interventions in fragile and conflict situations in the Middle East and North Africa region, Insights from Iraq, Lebanon, Republic of Yemen, and the West Bank and Gaza, 2020. https://elibrary.worldbank.org/doi/abs/10.1596/34036. (Accessed 4 May 2024).

[69] Hwang I.H., Yoon S. (2021). Electrification, labor force participation, and perceived social status for women in rural China. Asian J Women Stud.

[70] Vinci S., Nagpal D., Hodges T., Ferroukhi R., Accelerating off-grid renewable energy, IOREC 2014, Second international off-grid renewable energy conference, Key findings and recommendations, 2015. https://iorec.irena.org/-/media/Files/IRENA/IOREC/2014/iorec_2014_key_findings.pdf. (Accessed 4 May 2024).

[71] Ferroukhi R., Hawila D., García-Baños C., Renewable energy benefits: Decentralised solutions in the agri-food chain, 2016. https://www.irena.org/-/media/Files/IRENA/Agency/Publication/2016/IRENA_Decentralised_solutions_for_agrifood_chain_2016.pdf. (Accessed 4 May 2024).

[72] Ferroukhi R., Nagpal D., Parajuli B., Off-grid renewable energy solutions to expand electricity access: An opportunity not to be missed, 2019. https://www.irena.org/publications/2019/Jan/Off-grid-renewable-energy-solutions-to-expand-electricity-to-access-An-opportunity-not-to-be-missed. (Accessed 4 May 2024).

[73] ENERGIA, Scaling up energy access through women-led businesses, 2016. https://energia.org/assets/2017/03/WE-brochure_webversion.pdf. (Accessed 4 May 2024).

[74] UN Women, UN Environment Programme (UNEP) (2016).

[75] Harrison K., Adams T., Jose Campos M., Eskenazi S., Chebet A., Why off-grid matters, 2024. https://60decibels.com/insights/why-off-grid-energy-matters-2024/. (Accessed 4 May 2024).

[76] Pueyo A., Maestre M., Carreras M., Bawakyillenuo S., Ngoo G., Unlocking the benefits of productive uses of energy for women in Ghana, Tanzania and Myanmar, Research Report RA6,2019. https://energia.org/document/unlocking-the-benefits-of-productive-uses-of-energy-for-women-in-ghana-tanzania-and-myanmar/. (Accessed 25 December 2023).

[77] Ruggles M., Lenci L., Muller T., The ENERGIA gender and energy research programme: A short overview of the results, 2019. https://www.energia.org/assets/2020/03/Energia-News-March-2020.pdf. (Accessed 4 May 2024).

[78] Kooijman A., Corry J., Ireri M., The role of appliances in achieving gender equality and energy access for all, 2020. https://energia.org/document/policy-brief-the-role-of-appliances-in-achieving-gender-equality-and-energy-access-for-all/. (Accessed 4 May 2024).

[79] CLASP, Off-grid appliance market survey, 2020. https://www.clasp.ngo/research/all/off-grid-appliance-market-survey-2020-1/. (Accessed 4 May 2024).

[80] Soler A., Jæger J., Lecoque D., Women entrepreneurs as key drivers in the decentralised renewable energy sector, Best practices and innovative business models, 2020. https://www.ruralelec.org/wp-content/uploads/2023/11/Gender-Energy-Publication.pdf. (Accessed 4 May 2024).

[81] The World Bank Group, Firms with female participation in ownership (% of firms), 2023. https://data.worldbank.org/indicator/IC.FRM.FEMO.ZS. (Accessed 25 December 2023).

[82] Hasanbeigi A., Energy-efficiency improvement opportunities for the textile industry, 2010. https://www.energystar.gov/sites/default/files/buildings/tools/EE_Guidebook_for_Textile_industry.pdf. (Accessed 25 December 2023).

[83] International Renewable Energy Agency (IRENA), Solar heat for industrial processes, Technology brief, 2015. https://www.irena.org/-/media/Files/IRENA/Agency/Publication/2015/IRENA_ETSAP_Tech_Brief_E21_Solar_Heat_Industrial_2015.pdf. (Accessed 25 December 2023).

[84] International Finance Cooperation (IFC), The World Bank Group, Enterprise surveys indicator descriptions, 2023. https://www.enterprisesurveys.org/content/dam/enterprisesurveys/documents/Indicator-Description.pdf. (Accessed 25 December 2023).

[85] Hinrichs-Rahlwes R., Renné D., Hornung C., Musafer N., Gibb D., Kumar C.H., Mirowicz A., Weckend S., Åberg E., Tai K., Bhagirath A., Ferroukhi R., Companies in transition towards 100% renewables: Focus on heating and cooling, 2021. https://coalition.irena.org/-/media/Files/IRENA/Agency/Publication/2021/Feb/IRENA_Coalition_Companies_in_Transition_towards_100_2021.pdf. (Accessed 26 December 2023).

[86] Majumdar T., A growth path model: An entrepreneurship growth pathway for women in last-mile communities, 2020. https://energia.org/document/a-growth-path-model-an-entrepreneurship-growth-pathway-for-women-in-last-mile-communities/. (Accessed 4 May 2024).

[87] Kanase A.B., Patil A.N., Patil S.A., Patil V.J., Kodag S.V., Patil R.P., Pawar P.B. (2021). Analysis of efficiency with different GCV of biomass briquette and efficiency improvement opportunity in fire tube smoke type boiler. International Research Journal of Modernization in Engineering Technology and Science.

[88] Stiel A., Skyllas-Kazacos M. (2012). Feasibility study of energy storage systems in wind/diesel applications using the HOMER model. Appl. Sci..

[89] Habib G., Venkataraman C., Shrivastava M., Banerjee R., Stehr J.W., Dickerson R.R. (2004). New methodology for estimating biofuel consumption for cooking: atmospheric emissions of black carbon and sulfur dioxide from India. Global Biogeochem. Cycles.

[90] Islam A., Roy H., Rahman M.M. (2022). Energy efficiency study of household natural gas burner using pot-bottom shield and modified pot arrangement. Energy Rep..

[91] Ekouedjen E.K., Fagbemi L.A., Zannou-Tchoko S.J., Bakounoure J. (2020). Energy performance, safety and durability of charcoal cooking stoves commonly used in West Africa: Benin case study. AIMS Energy.

[92] Ballard-Tremeer G., Jawurek H.H. (1996). Comparison of five rural, wood-burning cooking devices: Efficiencies and emissions. Biomass Bioenergy.

[93] Tash, Fabric blog: Understanding fabric weight in order to choose the right fabric, 2015. https://blog.fabricuk.com/understanding-fabric-weight/. (Accessed 6 February 2024).

[94] Nepomuceno R.C., Watanabe P.H., Freitas E.R., Cruz C.E.B., Peixoto M.S.M., De Sousa M.L. (2014). Quality of quail eggs at different times of storage. Ciencia Anim. Bras..

[95] Njoro J.N., Community initiatives in livestock improvement: The case of Kathekani, Kenya, 2003. https://www.fao.org/3/Y3970E/y3970e07.htm#TopOfPage (accessed February 6, 2024).

[96] Kubkomawa H.I. (2017). Indigenous breeds of cattle, their productivity, economic and cultural values in sub-saharan Africa: A review. International Journal of Research Studies in Agricultural Sciences (IJRSAS).

[97] Olayemi F.F., Adedayo M., Bamishaiye R., Iyabo E., Fidelis A., Proximate composition of catfish (Clarias gariepinus), Int. J. Fish. Aquacult. 3 (2011) 96–98. https://www.researchgate.net/publication/267715900_Proximate_composition_of_catfish_Clarias_gariepinus_smoked_in_Nigerian_stored_products_research_institute_NSPRI_Developed_kiln. (Accessed 6 February 2024).

[98] Central Bank of Egypt, Exchange rates historical data, 2023. https://www.cbe.org.eg/en/economic-research/statistics/exchange-rates/historical-data. (Accessed 26 December 2023).

[99] Central Bank of Ghana, Monthly exchange rates indicators, 2023. https://www.bog.gov.gh/economic-data/exchange-rate/. (Accessed 26 December 2023).

[100] Central Bank of Kenya, Foreign exchange rates, 2023. https://www.centralbank.go.ke/rates/forex-exchange-rates/. (Accessed 26 December 2023).

[101] Xe, US Dollar to Malawian Kwacha exchange rate chart, 2023. https://www.xe.com/currencycharts/?from=USD&to=MWK&view=5Y. (Accessed 26 December 2023).

[102] Central Bank of Nigeria, Exchange rates, 2023. https://www.cbn.gov.ng/rates/exchratebycurrency.asp. (Accessed 26 December 2023).

[103] Xe, US Dollar to Tanzanian Shilling exchange rate chart, 2023. https://www.xe.com/currencycharts/?from=USD&to=TZS&view=2Y. (Accessed 26 December 2023).

[104] Central Bank of Tunisia, Monetary, economic and financial statistics, 2023. https://www.bct.gov.tn/bct/siteprod/cours.jsp?la=AN. (Accessed 26 December 2023).

[105] The World Bank Group, Africa electricity grids explorer, 2017. https://africagrid.energydata.info/. (Accessed 29 March 2024).

[106] The World Bank Group, International Energy Agency (IEA), International Renewable Energy Agency (IRENA), United Nations Statistics Division (UNSD), Tracking SDG 7: The energy progress report, 2023. https://trackingsdg7.esmap.org/data/files/download-documents/sdg7-report2023-full_report.pdf. (Accessed 1 April 2024).

[107] Hunt V., Layton D., Prince S., Why diversity matters, 2015. https://www.mckinsey.com/capabilities/people-and-organizational-performance/our-insights/why-diversity-matters. (Accessed 28 February 2024).

[108] Gad D., Leone P., Dataset: Raw data to paper “Productive use of energy of women-owned micro-, small-, and medium-sized enterprises: Insights from food and textile businesses in selected African countries”, Mendeley Data, V1 (2024). 10.17632/pmrxfs59mr.1. (Accessed 22 May 2024). PMC11219329 38961915

[109] Cahyani A.D., Nachrowi N.D., Hartono D., Widyawati D. (2022). Between insufficiency and efficiency: Unraveling households’ electricity usage characteristics of urban and rural Indonesia. Energy for Sustainable Development.

